# The In Vivo Existence Forms of Engeletin and Their Anti-Hyperuricemia Activity

**DOI:** 10.3390/ijms27125353

**Published:** 2026-06-13

**Authors:** Yang Lv, Jing Zhang, Shao-Jing Chen, Jing Zhang, Xing Han, Ming-Ying Shang, Guang-Xue Liu, Xuan Wang, Shao-Qing Cai, Feng Xu

**Affiliations:** 1 State Key Laboratory of Natural and Biomimetic Drugs, School of Pharmaceutical Sciences, Peking University, Beijing 100191, China; ly1334@126.com (Y.L.); 2011210080@stu.pku.edu.cn (J.Z.); zhangjing823@yeah.net (J.Z.); 2011110096@stu.pku.edu.cn (X.H.); 2 Division of Pharmacognosy, School of Pharmaceutical Sciences, Peking University Health Science Center, Beijing 100191, China; shaojing_chen@163.com (S.-J.C.); myshang@bjmu.edu.cn (M.-Y.S.); guangxl@bjmu.edu.cn (G.-X.L.); 3 School of Pharmaceutical Sciences, Zhejiang Chinese Medical University, Hangzhou 310053, China; 4 Department of Chemical Biology, Peking University Health Science Center, Beijing 100191, China; xuanwang6818@bjmu.edu.cn

**Keywords:** engeletin, metabolite, smilacis glabrae rhizoma, hyperuricemia

## Abstract

Smilacis Glabrae Rhizoma is a traditional Chinese medicine commonly used for hyperuricemia. Engeletin, one of its major flavonoids, exhibits various pharmacological activities, but its in vivo uric acid-lowering activity and metabolic processes remain unclear. This study aims to elucidate the in vivo existence forms of engeletin and the pharmacological basis underlying its uric acid-lowering effects. The in vivo metabolites of engeletin were identified by using UHPLC-Q-TOF-MS. The xanthine oxidase inhibitory activity was investigated using in vitro enzymatic assays. The in vivo uric acid-lowering effect was evaluated in hyperuricemic mice. A total of 11, 34, 7, 6, and 5 compounds were detected in urine, feces, serum, liver, and kidney samples, respectively. After removing duplicates, 52 compounds were preliminarily identified as in vivo existence forms of engeletin. The main metabolic reaction types were glucuronidation, sulfation, and hydrolysis. Engeletin exhibited no xanthine oxidase inhibitory activity in vitro but possessed uric acid-lowering activity in vivo. Neoisoastilbin and naringenin were metabolites with both xanthine oxidase inhibitory activity and uric acid-lowering activity. The in vivo uric acid-lowering activity of engeletin may be attributable to its two metabolites rather than itself. This study elucidated the pharmacological basis of engeletin and laid the foundation for developing potential therapeutics for hyperuricemia.

## 1. Introduction

In recent years, there has been an upward trend in the global prevalence of hyperuricemia and gout, with an earlier onset age in younger demographic [1,2]. Hyperuricemia is a prerequisite and primary risk factor for gout development [3]. Research indicates a clear, concentration-dependent relationship between serum urate levels and the incidence of gout [4]. Additionally, hyperuricemia is associated with various metabolic disorders, such as diabetes, hypertension, and kidney disease [5,6].

Hyperuricemia generally occurs due to an imbalance in uric acid homeostasis caused by excessive uric acid production or insufficient uric acid excretion [7]. Currently, the drugs used clinically to treat hyperuricemia mainly include allopurinol and febuxostat which function as inhibitors of xanthine oxidase [8], as well as benzbromarone and lesinurad which facilitate the excretion of uric acid [9].

Despite their efficacy in reducing uric acid levels, the adverse reactions of allopurinol [10], febuxostat [11] or benzbromarone [12] limit their widespread use. Consequently, the quest for novel agents to address hyperuricemia has emerged as a research priority.

Natural products have demonstrated substantial efficacy in the treatment of hyperuricemia. For instance, naringenin has been reported to inhibit xanthine oxidase in vitro [13], and oral administration of 50 mg/kg naringenin significantly reduced serum uric acid levels in hyperuricemic model mice [14]. Quercetin also exhibits considerable xanthine oxidase inhibitory activity [15], and doses of 50 mg/kg or more have the capacity to inhibit hepatic xanthine oxidase activity and significantly reduce serum uric acid concentrations in potassium oxonate-induced hyperuricemic mice [16].

Smilacis Glabrae Rhizoma is one of the commonly used traditional Chinese medicines for treating hyperuricemia [17,18], and current research indicates that its uric acid-lowering activity is related to its flavonoid content [19,20]. Engeletin (dihydrokaempferol 3-rhamnoside, CAS Registry Number: 572-31-6) is a flavonoid glycoside which presents in higher content in Smilacis Glabrae Rhizoma (0.85 mg/g) and Smilacis Chinae Rhizoma (3.5 mg/g) [21]. Engeletin has various pharmacological activities, such as anti-inflammatory [22], antioxidant [23], and antitumor [24] effects. Although one study suggests that engeletin may be associated with the uric acid-lowering activity of Smilacis Glabrae Rhizoma [25], there are no direct studies indicating whether engeletin can exert any effects related to uric acid-lowering activity in vivo. Furthermore, a single flavonoid can be biotransformed into more than one hundred metabolites in vivo [26]. The absence of reports on the metabolic processes of engeletin in vivo hinders the determination of whether its effective forms are itself, its active metabolites, or a combination of both.

Determining the effective forms of natural products is a vital preliminary step for lead compound discovery and subsequent drug development. Elucidating the metabolic process of engeletin and connecting its existence forms with pharmacological effects may help identify which compounds are actually responsible for urate reduction and xanthine oxidase inhibitory activity. This is vital for not only elucidating the pharmacodynamic basis of engeletin, but also for selecting candidate molecules for further pharmacological evaluation, structural optimization and development as potential therapeutic agents for hyperuricaemia.

The primary objective of this study is to investigate the metabolic process of engeletin in vivo by using UHPLC-Q-TOF-MS to identify its existence forms, and to screen and find its effective forms through xanthine oxidase inhibition assays in vitro and hyperuricemia animal model in vivo. This study provides crucial scientific evidence for elucidating the pharmacological basis of engeletin, and also offers insights into the relationship between the in vivo processes and biological activity of natural products.

## 2. Results and Discussion

The base peak chromatograms (BPCs) of negative and positive ion modes of urine samples from the preliminary experiments are shown in Figure S1 as an example. Upon comparison, we found that the negative ion mode provided more abundant ionic information. In contrast, under the positive ion mode, the signal responses of the metabolites were relatively weak, and the fragmentation patterns of many ions were not sufficiently informative. Therefore, the negative ion mode was ultimately selected as the primary analytical mode for the subsequent analysis.

By comparing the BPC profiles of samples (Figures S2–S4) from the control group and the engeletin-treated group, and combining this with the neutral loss pattern, 11, 33, 6, 6, and 5 metabolites of engeletin were identified in drug-containing urine, feces, serum, liver, and kidneys, respectively. Additionally, the parent compound was detected in the drug-containing feces and serum samples.

Excluding identical metabolites, a total of 51 metabolites of engeletin were identified (Figures S5–S11). The in vivo existence forms included both metabolites and the parent compound. After combining 51 metabolites with one parent compound, a total of 52 compounds were identified as in vivo existence forms. The LC-MS data of engeletin and its metabolites are shown in Table 1 and Table S1, and the major metabolites were characterized.

### 2.1. The Fragmentation Pathways of Engeletin

**Figure 1:**
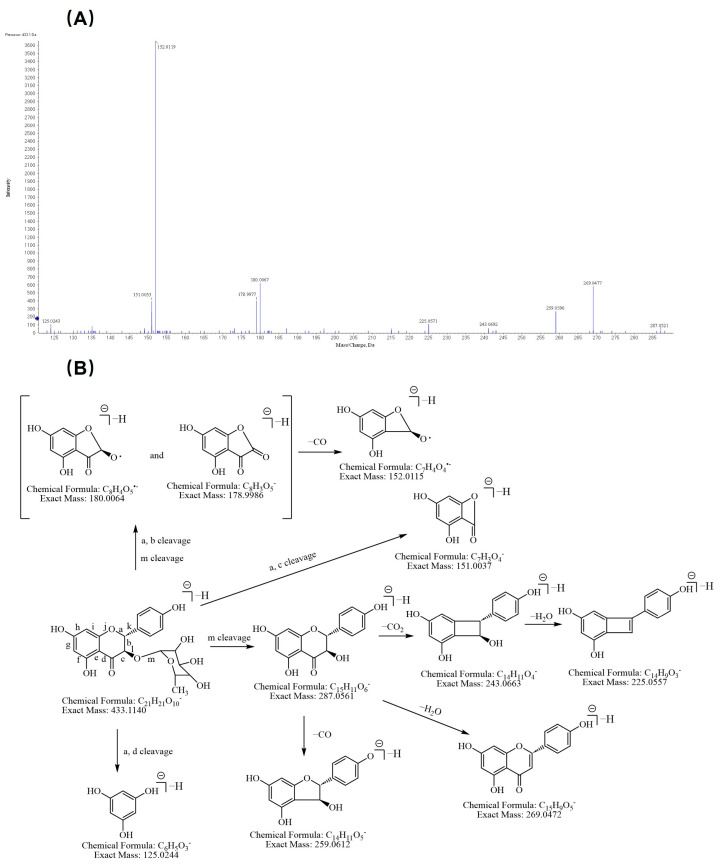
The MS/MS spectra in negative ion mode and proposed fragmentation pathways of engeletin. (**A**) The MS/MS spectra of engeletin in negative ion mode; (**B**) the proposed fragmentation pathways of engeletin. To conveniently describe the fragment characteristics, the bonds in the skeleton were designated by the letters a–m.

The proposed fragmentation pathways of engeletin are depicted in Figure 1. In the negative ion mode, engeletin showed [M−H]^−^ at *m*/*z* 433.1140 (C_21_H_21_O_10_). The ion with *m*/*z* 433.1140 loses 146.06 Da (C_6_H_10_O_4_) and 18.01 Da (H_2_O) consecutively to form fragment ions with *m*/*z* 287.0521 and *m*/*z* 269.0477, respectively. Additionally, a fragment ion with *m*/*z* 259.0596 was observed, which was formed by the loss of 27.99 Da (CO) from the parent ion with *m*/*z* 287.0521.

### 2.2. Detection and Identification of the Metabolites of Engeletin In Vivo

#### 2.2.1. Metabolites with the Skeleton of Engeletin

##### Engeletin (E0) and Its Isomer (E1–E5)

The [M−H]^−^ of compounds E0–E5 was detected at *m*/*z* 433.11, and their molecular formula was deduced as C_21_H_22_O_10_. Compound E0 was identified as engeletin due to its retention time and mass spectrometric fragmentation pattern being consistent with the engeletin reference compound. In the secondary mass spectra of E1–E5, fragment ions at *m*/*z* 269.04, *m*/*z* 259.06, *m*/*z* 180.01, and *m*/*z* 152.01, which were identical to those of engeletin, were all observed. However, their retention times differed from that of engeletin, and thus they were identified as isomers of engeletin.

##### Metabolites Formed by Sulfation of Engeletin (E6–E10)

The [M−H]^−^ of compounds E6–E10 was detected at *m*/*z* 513.07, with their molecular formula deduced as C_21_H_22_O_13_S. In the MS^2^ spectra of E8–E10, a fragment ion at *m*/*z* 433.11 (C_21_H_21_O_10_) was observed, which was formed by the loss of 79.96 Da (SO_3_) from the precursor ion at *m*/*z* 513.07. Their MS^2^ spectra also showed fragment ions at *m*/*z* 269.04, *m*/*z* 180.01, and *m*/*z* 152.01, which were consistent with those of engeletin. Thus, E8–E10 were identified as engeletin sulfates or isomers. In the MS^2^ spectra of E6 and E7, a fragment ion at *m*/*z* 431.10 (C_21_H_19_O_10_) was detected, resulting from the loss of 81.97 Da (H_2_SO_3_) from the ion at *m*/*z* 513.07. Given that the neutral loss of H_2_SO_3_ was observed in MS^2^ spectra of E6 and E7, it was postulated that the hydroxyl group linked with sulfuric acid was neither an enol hydroxyl nor part of a conjugated system. Moreover, a double bond would form to establish a conjugated system after the removal of the sulfate group.

Therefore, the hydroxyl groups at positions C-5, C-7, and C-4′ were less likely to be conjugated with sulfuric acid. When the hydroxyl group on glucosyl was sulfated and subsequent removal of the sulfate group occured, a carbonyl group was generated, which could form a conjugated system through keto-enol tautomerism. Hence, it was inferred that E6 and E7 underwent metabolic reactions involving the conjugation of glucosyl hydroxyl groups with sulfuric acid.

**Figure 2:**
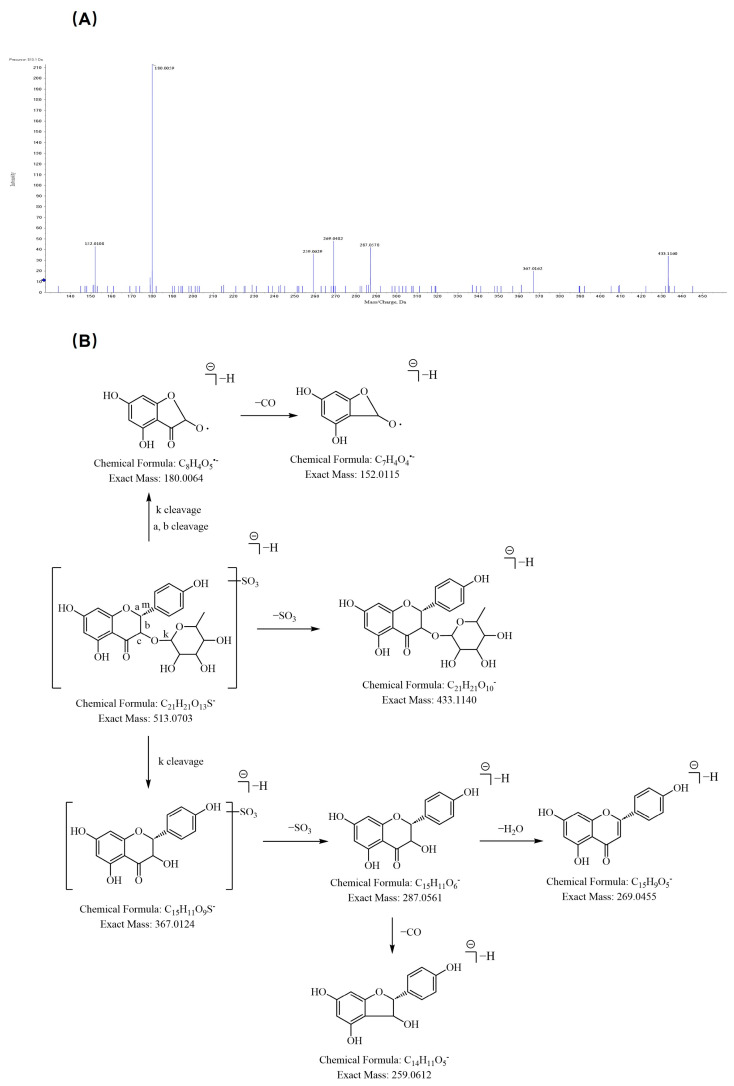
The MS/MS spectra in negative ion mode and proposed fragmentation pathways of E10. (**A**) The MS/MS spectra of E10 in negative ion mode; (**B**) the proposed fragmentation pathways of E10. To conveniently describe the fragment characteristics, the bonds in the skeleton were designated by the letters a, b, c, m, and k.

Since E6–E10 are isomers, we take E10 as a representative example; the potential fragmentation pathways of E10 are proposed and illustrated in Figure 2.

##### Metabolites Formed by Glucuronidation of Engeletin (E11–E22)

**Figure 3:**
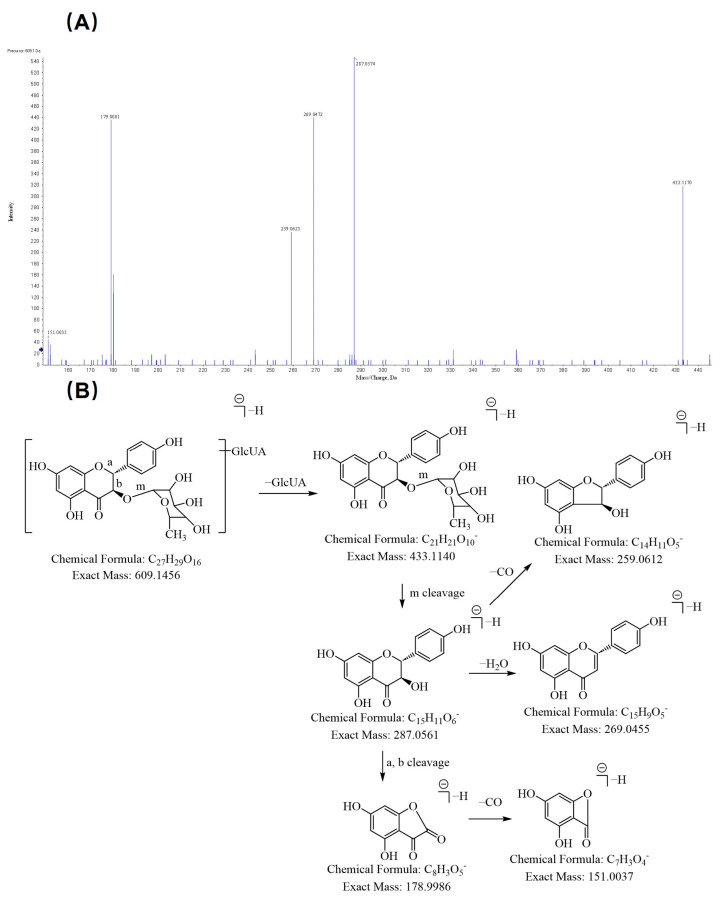
The MS/MS spectra in negative ion mode and proposed fragmentation pathways of E17. (**A**) The MS/MS spectra of E17 in negative ion mode; (**B**) the proposed fragmentation pathways of E17. To conveniently describe the fragment characteristics, the bonds in the skeleton were designated by the letters a, b, and m.

The [M−H]^−^ of compounds E11–E22 was detected at *m*/*z* 609.15, with their molecular formula deduced as C_27_H_30_O_16_. In the MS^2^ spectra of E11–E22, aglycone ions at *m*/*z* 433.11 (C_21_H_21_O_10_) were consistently observed, which were formed by the loss of 176.04 Da (C_6_H_8_O_6_) from the quasi-molecular ion at *m*/*z* 609.15. Furthermore, characteristic fragment ions of engeletin, including *m*/*z* 269.04 and *m*/*z* 259.06, were all detected in their MS^2^ spectra. Thus, E11–E22 were identified as engeletin or isomer glucuronides. Taking E17 as an example, its potential fragmentation pathways are proposed and shown in Figure 3.

##### Metabolites Formed by Hydrogenation and Glucuronidation of Engeletin (E23–E25)

The [M−H]^−^ of compounds E23–E25 was detected at *m*/*z* 611.16, with their molecular formula deduced as C_27_H_32_O_16_. In the MS^2^ spectra of E23–E25, aglycone ions at *m*/*z* 435.14 (C_21_H_23_O_10_) were consistently observed, formed by the loss of 176.02 Da (C_6_H_8_O_6_) from the quasi-molecular ion *m*/*z* 611.16. Comparison with the molecular formula of engeletin, it was revealed that the aglycone ion contains two additional hydrogen atoms. Consequently, the aglycone was identified as dihydro-engeletin, and E23–E25 were characterized as dihydro-engeletin glucuronides.

##### Metabolites Formed by Hydroxylation of Engeletin (E26–E28)

**Figure 4:**
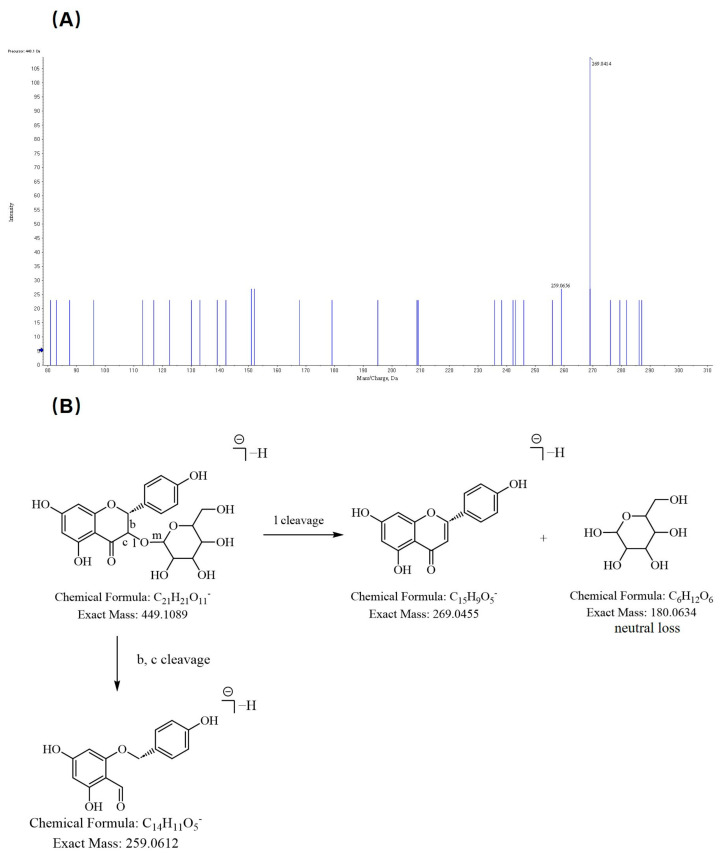
The MS/MS spectra in negative ion mode and proposed fragmentation pathways of E26. (**A**) The MS/MS spectra of E26 in negative ion mode; (**B**) the proposed fragmentation pathways of E26. To conveniently describe the fragment characteristics, the bonds in the skeleton were designated by the letters b, c, l, and m.

The [M−H]^−^ of compounds E26–E28 was detected at *m*/*z* 449.11, with their molecular formula predicted to be C_21_H_22_O_11_. Comparison with the molecular formula of engeletin revealed that E26–E28 contain one additional oxygen atom, indicating that they are hydroxylated engeletin. Since E26–E28 are isomers, we take E26 as a representative example; the potential cleavage pathways of E26–E28 were investigated. In its MS^2^ spectrum, a characteristic fragment ion at *m*/*z* 269.0414 was observed, which was generated by the loss of 180.06 Da (C_6_H_12_O_6_) from the quasi-molecular ion. This neutral loss is different from the neutral loss of 180.0064 Da in the mass spectra of engeletin, which suggests that the hydroxylation reaction may occur on the glycosyl moiety. Subsequently, the glycosidic bond was cleaved. The proposed fragmentation pathways of E26 are illustrated in Figure 4. The newly discovered metabolic pathway of flavonoid glycosides suggests that the body may further accelerate the excretion of engeletin by increasing the polarity of the glycoside. This is achieved specifically by adding a hydroxyl group to the methyl group at the C6 position of rhamnose.

##### Metabolites Formed by Methylation and Hydroxylation of Engeletin (E32–E35)

The [M−H]^−^ of compounds E32–E35 was detected at *m*/*z* 463.12, and their molecular formula was deduced as C_22_H_24_O_11_. When compared with the molecular formula of engeletin, it was found that E32–E35 had one additional carbon atom, two more hydrogen atoms, and one extra oxygen atom. Thus, it was postulated that E32–E35 were methyl-hydroxy-engeletin derivatives.

##### Metabolites Formed by Demethylation of Engeletin (E36)

**Figure 5:**
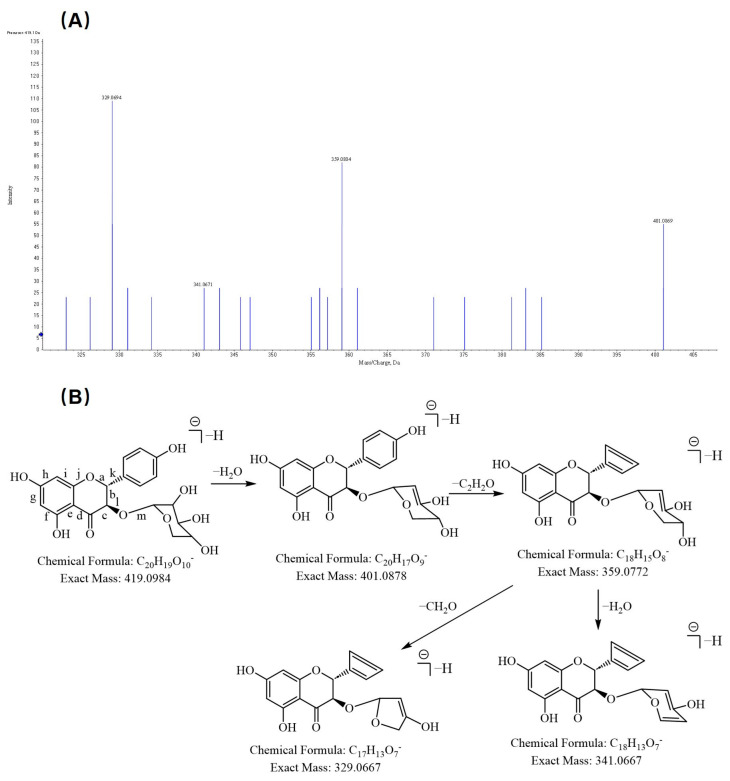
The MS/MS spectra in negative ion mode and proposed fragmentation pathways of E36. (**A**) The MS/MS spectra of E36 in negative ion mode; (**B**) the proposed fragmentation pathways of E36. To conveniently describe the fragment characteristics, the bonds in the skeleton were designated by the letters a–m.

The [M−H]^−^ of E36 was detected at *m*/*z* 419.0976, and its molecular formula was deduced to be C_20_H_20_O_10_. Comparison with the molecular formula of engeletin revealed that E36 contained one less carbon atom and two fewer hydrogen atoms, leading to the postulation that it was demethyl-engeletin. In the MS^2^ spectrum of E36, fragment ions such as *m*/*z* 401.0869 and *m*/*z* 359.0804 were observed, which were generated by the successive loss of 18.01 Da (H_2_O) and 42.01 Da (C_2_H_2_O) from the quasi-molecular ion. The possible fragmentation pathways of E36 are shown in Figure 5.

##### Metabolites Formed by Methylation of Engeletin Skeleton (E29–E31)

The [M−H]^−^ of E29 was detected at *m*/*z* 447.1285, with its molecular formula proposed as C_22_H_24_O_10_. In its MS^2^ spectrum, fragment ions were observed at *m*/*z* 269.0448, *m*/*z* 180.0063, and *m*/*z* 152.0104. Comparative analysis with the molecular formula of engeletin revealed that E29 contained one additional C atom and two extra H atoms, leading to the proposal that it was methyl-engeletin.

The [M−H]^−^ of E30 and E31 was detected at *m*/*z* 527.08, and their molecular formula was proposed to be C_22_H_24_O_13_S. In the MS^2^ spectra, fragment ions at *m*/*z* 445.11 (C_22_H_22_O_10_) were observed, which were generated by the loss of 81.97 Da (H_2_SO_3_) from the quasi-molecular ion. When compared with the molecular formula of E29, E30 and E31 were found to contain an additional SO_3_ moiety, and thus they were proposed to be methyl-engeletin sulfates.

##### Metabolites Formed by Methylation and Dehydrogenation of Engeletin (E37 and E38)

The [M−H]^−^ of E37 and E38 was detected at *m*/*z* 445.11, and their molecular formula was predicted to be C_22_H_22_O_10_. Comparison with the molecular formula of E29 revealed that E37 and E38 contained two fewer hydrogen atoms, leading to the postulation that they were methyl-dehydrogenated-engeletin derivatives. In the MS^2^ spectrum of E37, fragment ions such as *m*/*z* 299.0599 and *m*/*z* 281.0480 were observed, while in the MS^2^ spectrum of E38, fragment ion *m*/*z* 281.0479 was detected.

##### Metabolites Formed by Acetylation and Hydrogenation of Engeletin (E39)

The [M−H]^−^ of E39 was observed at *m*/*z* 477.1397, with its molecular formula proposed as C_23_H_26_O_11_. Comparative analysis with the molecular formula of engeletin revealed that E39 contained two additional C atoms, four extra H atoms, and one more O atom. It might have undergone acetylation and hydrogenation, and therefore it was acetylated-hydrogenated-engeletin.

#### 2.2.2. Metabolites with the Skeleton of Dihydrokaempferol (E40–E51)

**Figure 6:**
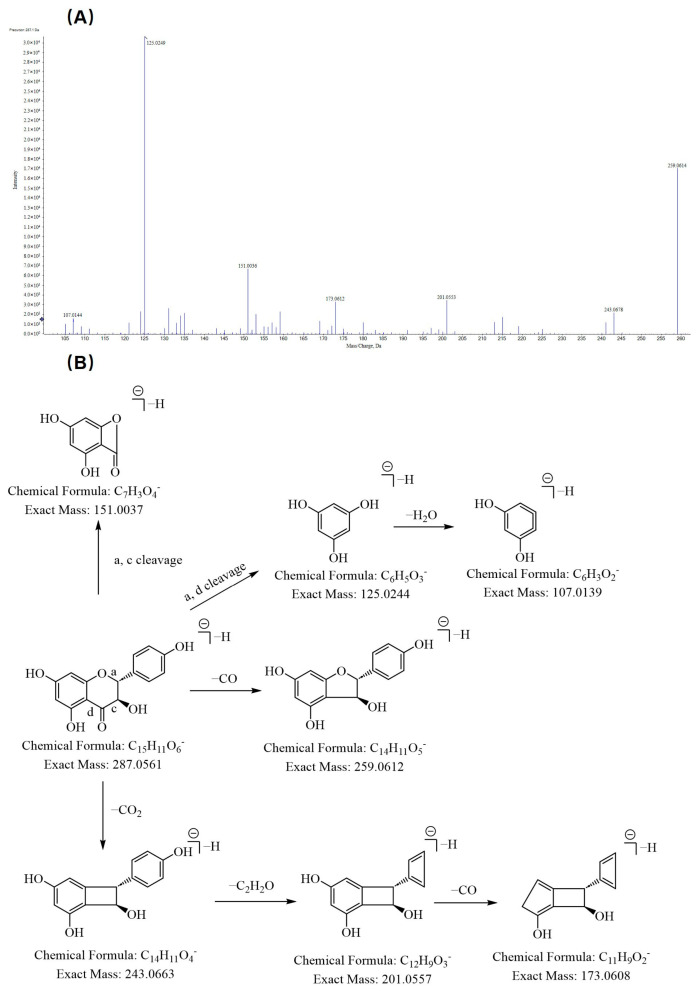
The MS/MS spectra in negative ion mode and proposed fragmentation pathways of E40. (**A**) The MS/MS spectra of E40 in negative ion mode; (**B**) the proposed fragmentation pathways of E40. To conveniently describe the fragment characteristics, the bonds in the skeleton were designated by the letters a, c, and d.

The [M−H]^−^ of E40 and E41 was detected at *m*/*z* 287.05, with their molecular formula predicted as C_15_H_12_O_6_. Using E40 as a representative, the fragmentation pathways of both E40 and E41 were proposed. In the MS^2^ spectrum of E40, fragment ions including *m*/*z* 259.0614 and *m*/*z* 243.0678 were observed, which were generated by the loss of 27.99 Da (CO) and 43.99 Da (CO_2_) from the quasi-molecular ion, respectively. Its retention time and mass spectral fragments matched those of the dihydrokaempferol reference compound, and thus it was identified as dihydrokaempferol. Since E41 was an isomer of E40, it was identified as a dihydrokaempferol isomer. We take E40 as a representative example; the proposed fragmentation pathways of E40 are illustrated in Figure 6.

**Figure 7:**
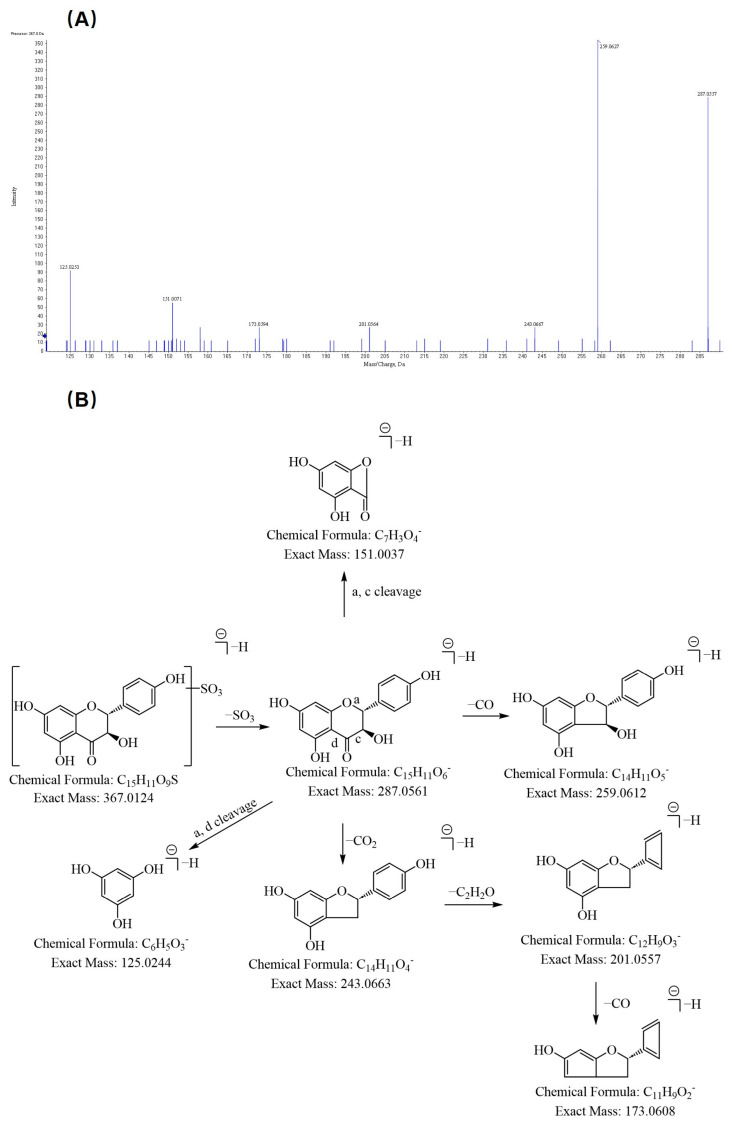
The proposed MS/MS spectra in negative ion mode and fragmentation pathways of E43. (**A**) The MS/MS spectra of E43 in negative ion mode; (**B**) the proposed fragmentation pathways of E43. To conveniently describe the fragment characteristics, the bonds in the skeleton were designated by the letters a, c, and d.

The [M−H]^−^ of E42–E45 was detected at *m*/*z* 367.01, and their molecular formula was proposed to be C_15_H_12_O_9_S. In their MS^2^ spectra, the loss of 79.96 Da (SO_3_) from the precursor ion yielded the same ion *m*/*z* 287.05 (C_15_H_11_O_6_). Therefore, E42–E45 were proposed to be dihydrokaempferol sulfates. Since E42–E45 are isomers, we take E43 as an example to explore the fragmentation pathways of E42–E45; the proposed fragmentation pathways of E43 are shown in Figure 7.

The [M−H]^−^ of E46 was detected at *m*/*z* 369.0269, with its molecular formula proposed as C_15_H_14_O_9_S. In its MS^2^ spectrum, an ion at *m*/*z* 289.0713 was observed, which was generated by the loss of 79.96 Da (SO_3_) from the quasi-molecular ion. Comparative analysis with the molecular formula of E42–E45 revealed that E46 contained two additional H atoms, leading to the proposal that it was hydrogenated-dihydrokaempferol sulfate.

The [M−H]^−^ of E47 was detected at *m*/*z* 449.1082, and its molecular formula was proposed as C_21_H_22_O_11_. In its MS^2^ spectrum, an aglycone ion at *m*/*z* 285.0375 (C_15_H_9_O_6_) was observed, which was generated by the loss of 164.07 Da (C_6_H_12_O_5_) from the quasi-molecular ion. By comparing with the molecular formula of E40 and E41, the aglycone ion was found to contain two fewer H atoms. Thus, the aglycone was proposed to be kaempferol, and E47 was proposed to be dihydrokaempferol glucoside.

The [M−H]^−^ of E48 was detected at *m*/*z* 303.0505, with its molecular formula proposed as C_15_H_12_O_7_. Since the mass spectral information of E48 matched that of the taxifolin reference compound, it was identified as taxifolin.

The [M−H]^−^ of E49 and E50 was detected at *m*/*z* 271.06, and their molecular formula was proposed to be C_15_H_12_O_5_. The mass spectral information of E50 was consistent with that of the naringenin reference compound, so E50 was identified as naringenin. As E49 was an isomer of E50, it was identified as a naringenin isomer.

The [M−H]^−^ of E51 was detected at *m*/*z* 381.0271, with its molecular formula proposed as C_16_H_14_O_9_S. In the MS^2^ spectrum, a fragment ion at *m*/*z* 299.0555 (C_16_H_11_O_6_) was observed, which was generated by the loss of 81.97 Da (H_2_SO_3_) from the quasi-molecular ion. Comparative analysis with the molecular formula of E42–E45 showed that the fragment ion at *m*/*z* 299.0555 (C_16_H_11_O_6_) contained one additional C atom and two extra H atoms. Therefore, E51 was proposed to be methyl-dihydrokaempferol sulfate.

### 2.3. Proposed Metabolic Pathways of Engeletin In Vivo

**Figure 8:**
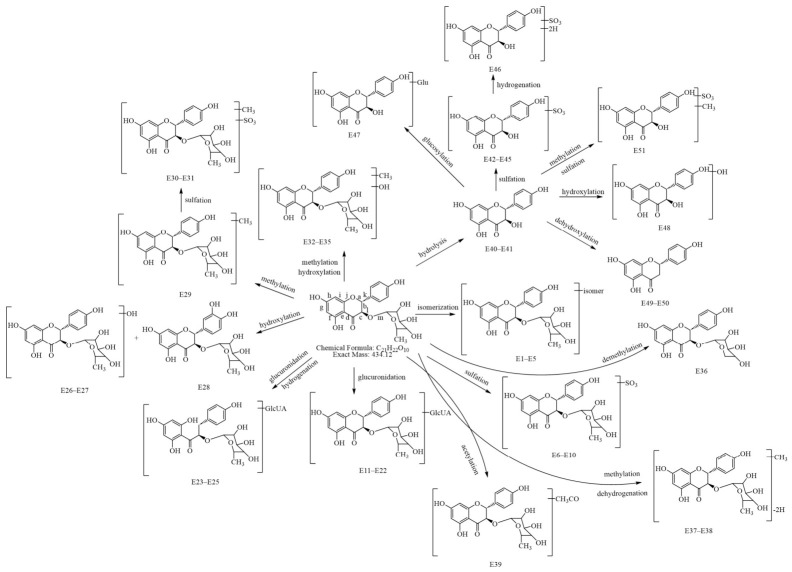
The proposed metabolic pathways of engeletin in mice. To conveniently describe the fragment characteristics, the bonds in the skeleton were designated by the letters a–m.

A total of 51 engeletin metabolites were identified from blood, urine, feces, kidney, and liver samples of mice administered with engeletin. Based on the structures of these metabolites, the potential metabolic pathways of engeletin in mice were deduced, as shown in Figure 8.

### 2.4. Metabolic Reaction Types of Engeletin in Mice

Among the metabolites of engeletin, 16 are phase I metabolites and 35 are phase II metabolites. Analysis of their structures indicates that the main in vivo reactions of engeletin are glucuronic acid conjugation, hydrolysis, methylation, and sulfation. The details are as follows:

From engeletin to its metabolites, 15 metabolites have undergone glucuronidation; 13 have undergone sulfation; 11 have undergone hydrolysis; 10 have undergone methylation; 8 have undergone hydroxylation; 5 have undergone isomerization; 4 have undergone hydrogenation; 2 have undergone dehydrogenation; 2 have undergone dehydroxylation; 1 has undergone demethylation; 1 has undergone glycosylation; and 1 has undergone acetylation (Table 2).

Among the 51 identified metabolites, 16 are discovered potential new compounds which are classified into four major categories: engeletin glucuronide, methyl-engeletin, methyl-engeletin sulfate, and acetylated-hydrogenated-engeletin. Among these, engeletin glucuronide features a highly polar glucuronic acid group introduced into the engeletin structure, imparting a negative charge at physiological pH. This glucuronide conjugation is a well-established phase II metabolic pathway that enhances hydrophilicity [27]. This structure confers the potential for active transport via organic anion transporters [28]. Therefore, engeletin glucuronides may exhibit altered membrane permeability and enhanced transporter-mediated disposition compared with the parent compound. For flavonoids and other polyphenols, methylation has been reported to increase lipophilicity and improve metabolic stability and membrane permeability in several cases [29]. Methyl-engeletin, formed by replacing the reactive phenolic hydroxyl group in engeletin with a methoxy group, may exhibit improved metabolic stability. Sulfation is another major phase II conjugation reaction that generally increases polarity and introduces an anionic group at physiological pH, thereby favoring aqueous solubility and transporter-mediated excretion [30,31]. Methyl-engeletin sulfate possesses a dual-modified structure. This combination of semi-polar and semi-nonpolar characteristics may represent a novel aspect of the compound. The acetyl group can increase the molecule’s lipophilicity and improve membrane permeability [32,33], while hydrogenation may further stabilize its structure. Consequently, acetylated-hydrogenated-engeletin may alter the original bioavailability of engeletin, conferring improved tissue permeability.

### 2.5. Xanthine Oxidase Inhibitory Activity of Engeletin and Its Metabolites

**Figure 9:**
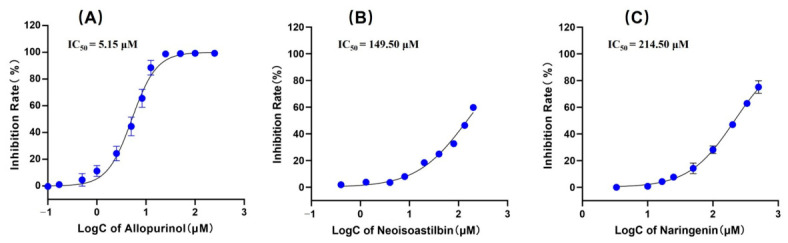
Inhibition rate of allopurinol, neoisoastilbin and naringenin on xanthine oxidase. (**A**) Allopurinol; (**B**) neoisoastilbin; (**C**) naringenin. Data are represented as mean ± SD (*n* = 3).

The in vitro xanthine oxidase inhibitory activity of engeletin and its four metabolites (neoisoastilbin, dihydrokaempferol, taxifolin, and naringenin) was investigated, with allopurinol as a positive control. The results demonstrated that allopurinol exhibited strong xanthine oxidase inhibitory activity, with an IC_50_ value of 5.15 μM, which is close to the previously reported value of 6.77 μM [34]. Neoisoastilbin and naringenin exhibited xanthine oxidase inhibitory activity, with IC_50_ values of 149.50 μM and 214.50 μM, respectively, whereas engeletin, dihydrokaempferol, and taxifolin exhibited no xanthine oxidase inhibitory activity with IC_50_ values. The xanthine oxidase inhibitory activity of neoisoastilbin is found for the first time. The inhibitory activity of naringenin is also generally consistent with the range of previous studies [13,35]. The results are shown in Figure 9A–C and Figure S12.

### 2.6. Molecular Docking Analysis

Molecular docking is a structure-based design method widely used in drug discovery and development [36,37,38]. It enables the identification of compounds with therapeutic potential, predicts interactions between targets and ligands or structure–activity relationships at the molecular level [39], and estimates binding energies. Therefore, molecular docking has become an essential tool for understanding compound–target interactions and supporting the development of novel therapeutic agents. Building on the identification of two “effective forms” of engeletin with xanthine oxidase inhibitory activity, molecular docking techniques were employed to investigate their binding sites and mechanisms of interaction with xanthine oxidase, with the aim of further elucidating the mechanisms underlying their pharmacological effects at the atomic and group levels. The binding energies and binding sites of the two active metabolites and allopurinol with xanthine oxidase are detailed in Table S2.

**Figure 10:**
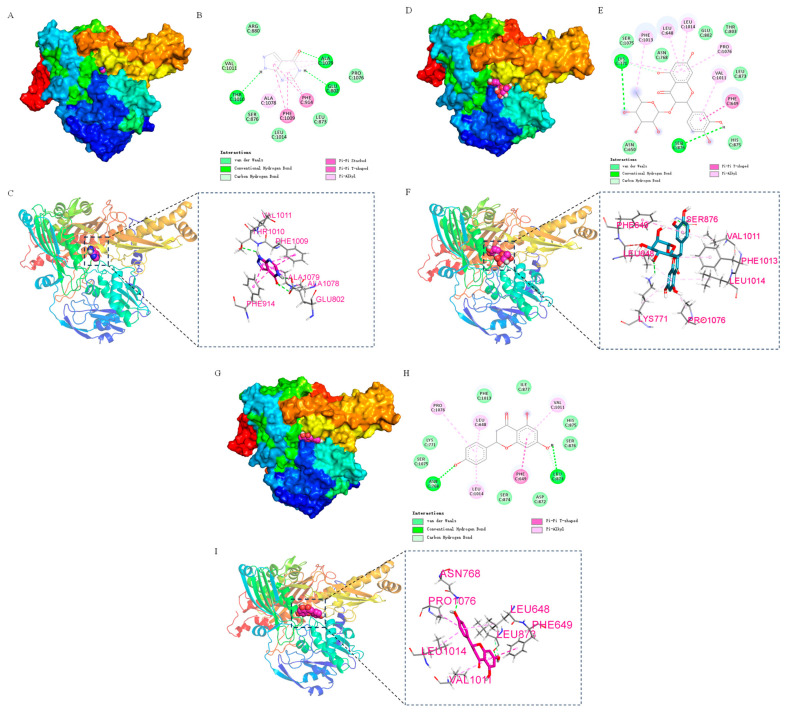
Molecular docking analysis of compounds and xanthine oxidase. (**A**) Overall 3D docking diagram of allopurinol with xanthine oxidase; (**B**) 2D diagram of amino acid residue interactions between allopurinol and xanthine oxidase; (**C**) 3D diagram of amino acid residue interactions between allopurinol and xanthine oxidase; (**D**) overall 3D docking diagram of neoisoastilbin with xanthine oxidase; (**E**) 2D diagram of amino acid residue interactions between neoisoastilbin and xanthine oxidase; (**F**) 3D diagram of amino acid residue interactions between neoisoastilbin and xanthine oxidase; (**G**) overall 3D docking view of naringenin with xanthine oxidase; (**H**) 2D interaction diagram of amino acid residues between naringenin and xanthine oxidase; (**I**) 3D interaction diagram of amino acid residues between naringenin and xanthine oxidase. The different colors in (**A**), (**D**), and (**G**) represent different amino acids.

The docking result of allopurinol with xanthine oxidase is shown in Figure 10A–C. As shown in Figure 10A,C, allopurinol could act on the active site of the Mo-pt (Molybdopterin) domain of xanthine oxidase. The binding energy between the two was −6.27 ± 0.06 kcal/mol. As shown in Figure 10B, the 3-NH, 4-O, and 7-NH groups of allopurinol could form hydrogen bonds with the key amino acid residues GLU802, ALA1079, and THR1010 in the Mo-pt active center of the enzyme, while the pyrazole ring and pyrimidine ring of allopurinol could form π–π conjugations with PHE1009 and PHE914, as well as π–alkyl interactions with ALA1078 and ALA1079. These results are consistent with previous reports indicating that allopurinol can form hydrogen bonds with THR1010, ALA1079, and GLU802, and form π–π conjugations with PHE1009 and PHE914 [40,41]. Therefore, hydrogen bonding and hydrophobic interactions were the primary interaction mechanisms between allopurinol and xanthine oxidase, leading to the formation of a stable complex between the two.

The docking results of neoisoastilbin with xanthine oxidase are shown in Figure 10D–F. As shown in Figure 10D,F, the neoisoastilbin could act on the active center of the Mo-pt domain of xanthine oxidase, with a binding energy of −6.90 ± 0.10 kcal/mol. As shown in Figure 10E, the rhamnose moiety and the 3′-OH group on the B ring of the neoisoastilbin could form hydrogen bonds with the key amino acid residues LYS771 and SER876 in the active center of the Mo-pt domain. The B ring could form a π–π conjugation with PHE649. The A ring, B ring, and rhamnose moiety could form π–alkyl interactions with LEU648, LEU1014, PRO1076, LYS771, VAL1011, and PHE1013. The 5-OH group on the A ring could form a carbon–hydrogen bond interaction with LYS771. Therefore, hydrogen bonding and hydrophobic interactions were the primary interaction types between neoisoastilbin and xanthine oxidase.

The docking results of naringenin with xanthine oxidase are shown in Figure 10G–I. As shown in Figure 10G,I, naringenin could act on the active center of the Mo-pt domain of xanthine oxidase, with a binding energy of −7.93 ± 0.06 kcal/mol. As shown in Figure 10H, the 7-OH group of the A ring and the 4′-OH group of the B ring of naringenin could form hydrogen bonds with the key amino acid residues LEU873 and ASN768 in the active center of the Mo-pt domain. The A ring could form a π–π conjugation with PHE649. The A and B rings could form π–alkyl interactions with VAL1011, PRO1076, LEU648, and LEU1014, respectively. The 7-OH group of the A ring could also form a carbon–hydrogen bond interaction with LEU873. Therefore, hydrogen bonding and hydrophobic interactions were the primary interaction types between naringenin and xanthine oxidase.

The Mo-pt domain serves as the active site for substrate binding and catalysis in xanthine oxidase [42]. This domain contains amino acid residues involved in catalysis, such as GLU802, as well as key amino acid residues involved in purine substrate recognition, such as PHE914, PHE1009, and THR1010. Furthermore, it includes amino acid residues that form the Mo-pt active site channel, such as LEU873 and VAL1011 [43,44]. The results of molecular docking indicate that both naringenin and neoisoastilbin can enter the Mo-Pt active site of xanthine oxidase and form potential interactions with key amino acid residues through hydrogen bonding and hydrophobic interactions. These findings reveal a dual inhibition mechanism of xanthine oxidase by the two effective forms, which not only directly bind to the catalytic site to interfere with the enzymatic reaction, but also block the binding of purine substrates by occupying the substrate entry channel, thereby effectively inhibiting xanthine oxidase activity.

Although neoisoastilbin and naringenin showed favorable binding interactions with xanthine oxidase in the molecular docking analysis, their inhibitory effects were weaker than that of the positive control allopurinol in both in vitro and in vivo assays. Notably, the activity order observed in the experimental was consistent, namely allopurinol > neoisoastilbin > naringenin. The discrepancy between docking prediction and experimental activity may be attributed to the limitations of molecular docking. Docking provides a theoretical prediction of ligand–protein interactions under static condition, whereas actual xanthine oxidase inhibition is affected by multiple factors, such as ligand solubility, stability and metabolism. Therefore, the docking results should be regarded as supportive evidence for possible interactions between these compounds and xanthine oxidase, rather than direct evidence of stronger inhibitory activity than allopurinol.

### 2.7. Effects of Engeletin and Its Metabolites on Serum Uric Acid Levels in Hyperuricemia Mice

**Figure 11:**
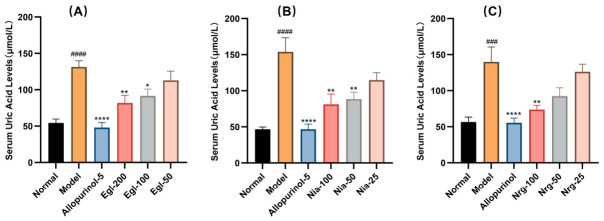
Effects of different doses of engeletin and its metabolites on serum uric acid levels in hyperuricemic model mice. (**A**) engeletin, Egl; (**B**) neoisoastilbin, Nia; (**C**) naringenin, Nrg. Data are represented as mean ± SEM (*n* = 8). ^####^ *p* < 0.0001, ^###^ *p* < 0.001, compared with the normal group. **** *p* < 0.0001, ** *p* < 0.01, * *p* < 0.05, compared with the model group.

We investigated whether engeletin and its two metabolites (neoisoastilbin and naringenin) have the ability to inhibit the formation of uric acid. The results showed that compared with the control group, serum uric acid levels in the model group were significantly elevated (*p* < 0.001 and *p* < 0.0001), indicating that the mice model was successfully established. The positive control drug allopurinol significantly reduced serum uric acid levels in the model mice (*p* < 0.0001). As shown in Figure 11B, both 50 mg/kg and 100 mg/kg doses of neoisoastilbin significantly reduced serum uric acid levels in model mice (*p* < 0.01). As shown in Figure 11C, a 100 mg/kg dose of naringenin significantly reduced serum uric acid levels in model mice (*p* < 0.01), which is consistent with a previous report that naringenin exhibited uric acid-lowering activity in vivo [14]. Interestingly, although engeletin did not exhibit inhibitory activity in the in vitro enzymatic assay, it demonstrated significant in vivo uric acid-lowering activity at doses of 100 mg/kg and 200 mg/kg (*p* < 0.05 and *p* < 0.01), as shown in Figure 11A. The uric acid-lowering activity of neoisoastilbin was firstly discovered. The above results indicate that engeletin might exert its uric acid-lowering effect in vivo through its metabolites rather than the compound itself. The pivotal function of the gut microbiota in the absorption and metabolism of flavonoids has been well documented [45,46,47]. Following ingestion, flavonoid glycosides are subject to deglycosylation by gut microbiota, resulting in the release of free aglycones [47,48]. These aglycones then undergo a series of reactions, leading to the formation of numerous metabolites [49]. These metabolites also interact with the gut microbiota, thereby regulating a range of biological activities [50,51]. Recent studies have shown that hyperuricemia is also associated with the gut microbiota [52,53,54,55]. Since naringenin and neoisoastilbin were detected as metabolites of engeletin in feces, it is possible that engeletin is partially transformed by the gut microbiota in vivo. These metabolites may primarily exert local effects in the intestinal tract, such as modulating the gut microbiota. In addition, a small fraction of these metabolites may be absorbed into the systemic circulation. Although the two metabolites were not detected in serum and liver samples, this does not exclude their potential contribution to the overall pharmacological effects, as low-abundance active metabolites may exert pharmacological effects through mixture effects [56]. Therefore, the metabolites of engeletin may contribute to its anti-hyperuricemic activity by regulating gut microbiota and, potentially, by participating in xanthine oxidase inhibition after absorption and further metabolism. However, further targeted pharmacokinetic studies are required to confirm their absorption, distribution, and contribution to xanthine oxidase inhibition in vivo. It can be inferred that the hypouricemic effect of engeletin is at least partially explained by the contribution of the hypouricemic effects of those two active metabolites. To further clarify the relationship between in vivo and in vitro pharmacological activities, more indicators should be investigated in future studies.

## 3. Materials and Methods

### 3.1. Reagents and Chemicals

Engeletin (batch No. R15N8F48275, purity ≥ 98%) was purchased from Yuanye Bio-Technology Co., Ltd. (Shanghai, China). Taxifolin (batch No. MUST-21120207, purity ≥ 98%), dihydrokaempferol (batch No. MUST-23052701, purity ≥ 98%) and naringenin (batch No. MUST-22072711, purity ≥ 98%) were bought from Must Bio-Technology Co., Ltd. (Chengdu, China). Neoisoastilbin (batch No. DSTDX007801, purity ≥98%) was acquired from Desite Bio-Technology Co., Ltd. (Chengdu, China). Xanthine oxidase (batch No. SLCG2160, 0.4 units/mg protein) and dimethyl sulfoxide (DMSO, batch No. 102515645) were purchased from Sigma-Aldrich (St. Louis, MO, USA). Xanthine was obtained from Aladdin Biochemical Technology Co., Ltd. (Shanghai, China). Phosphate-buffered saline (PBS, 0.05 M, pH 7.4) was purchased from Biorigin (Beijing, China). Formic acid, methanol and acetonitrile were acquired from Thermo Fisher Scientific (Waltham, MA, USA). Sodium carboxymethyl cellulose (CMC-Na) was purchased from Beijing MREDA Technology Co., Ltd. (Beijing, China). A uric acid (UA) Test Kit was purchased from Nanjing Jiancheng Bioengineering Institute (Nanjing, China). Ultrapure water was prepared using a Milli-Q Integral 3 ultrapure water machine (Millipore, Burlington, MA, USA).

### 3.2. Animals and Experimental Procedures

Male ICR mice (25–30 g) were purchased from the Department of Laboratory Animal Science, Peking University Health Science Center (Beijing, China, licensed ID: SCXK (Beijing) 2022-0009).

#### 3.2.1. Metabolic Study of Engeletin

Ten mice were randomly divided into two groups, the control group and engeletin-treated group, with five mice in each group. Prior to the experiment, all mice were placed in mouse metabolic cages for 3 days of acclimatization, during which normal feeding and drinking were ensured. The room temperature was maintained at 25 ± 5 °C and relative humidity at 40 ± 5%, with a 12 h light/dark cycle. Following the acclimatization period, oral administration was conducted for 7 consecutive days. The engeletin group received an oral dose of 100 mg/kg engeletin (dispersed in 0.5% CMC-Na solution) at 9:00 a.m. daily, while the control group received an equal volume of 0.5% CMC-Na solution. Water and food were provided regularly during the treatment period. All animal experiments in this study were approved by the Biomedical Ethics Committee of Peking University (approval number: LA2021269).

Subsequent to the initiation of drug administration to the mice, a comprehensive collection of urine and fecal samples was conducted over the course of the 7-day administration period. Prior to each urine collection, 10 mL of anhydrous ethanol was added to each urine collection tube with the objective of inhibiting bacterial growth. One hour after the final administration, blood was collected by enucleation of the eyes. After that, the blood was allowed to stand at 4 °C for 1 h. Then, it was subjected to centrifugation at 5000 rpm (2054× *g*) at 4 °C for 15 min. Thereafter, the superior portion was collected. Following the collection of blood, the liver and kidneys were promptly extracted and thoroughly rinsed with physiological saline until the fluid emanating from the surface or cavities was devoid of visible blood. All samples were subsequently stored at −80 °C.

Because the aim of this analysis was mainly to comprehensively identify the in vivo existence forms of engeletin rather than to quantitatively compare inter-individual differences, pooling samples has several advantages for this type of qualitative metabolite-profiling study. It can increase the overall abundance of trace metabolites, thereby improving the detection sensitivity and coverage of UHPLC-MS/MS analysis. It can also reduce the influence of individual biological variation and provide a more representative metabolic profile. Therefore, samples from the same group were pooled prior to analysis. The urine, feces, and serum samples from the blank group and the engeletin administration group were then combined to create a pooled sample for each group. The pooled urine samples were filtered and subsequently evaporated to dryness at 50 °C. Afterwards the samples were subjected to ultrasonication with methanol at a volume concentration of 10× (mL/g) for a duration of 30 min. The extract was centrifuged at 8000 rpm (6010× *g*) for a duration of 15 min. Thereafter, the supernatant was evaporated to dryness at 55 °C. Finally, a methanol solution at a concentration of 0.1 g/mL was made and then the mixture was subjected to ultrasonic dissolution. In order to proceed to the subsequent stage of the analysis, a 0.22 μm microporous membrane was required for the filtration process, following which the sample was stored at −80 °C.

The fecal samples from the blank group and the engeletin treatment group were combined respectively. Then the combined sample was dried at 50 °C for 48 h prior to grinding into powder. Subsequently, the samples were subjected to ultrasonic extraction with fivefold volume of methanol for 30 min, repeated three times. The extracts were pooled, subjected to centrifugation at 8000 r/min (6010× *g*) for 15 min, and the resulting supernatant was further evaporated to dryness at 50 °C. Finally, 5 mL/g of methanol was added for dissolution. The solution was filtered through a 0.22 μm microporous membrane, and stored at −80 °C for further analysis.

The pooled serum samples were subjected to ultrasonication with methanol at a volume concentration of 4× (mL/mL) for a duration of 30 min. The extract was centrifuged at 5000 rpm (2054× *g*) for a duration of 15 min, and the resulting supernatant was further evaporated to dryness under a nitrogen stream. Finally, 200 μL of methanol was added for dissolution. The solution was filtered through a 0.22 μm microporous membrane, and stored at −80 °C for further analysis.

Following the removal of residual moisture from each organ sample by filter paper, the organ samples were weighed. Then, the samples were immersed in purified water at a volume four times the weight of the organ sample (mL/g) to ensure homogenization. Subsequently, a tenfold volume of methanol was introduced, followed by sonication for 30 min. The extract was then centrifuged at 5000 rpm (2054× *g*) for 15 min. The upper layer was collected and concentrated to dryness under reduced pressure. The dry extracts from each organ sample were weighed equally and redissolved in 2 mL of methanol. Then, the extracts were filtered through a 0.22 μm membrane before proceeding to analysis.

#### 3.2.2. Validation of Uric Acid-Lowering Activity of Engeletin and Its Metabolites In Vivo

Male ICR mice purchased from the Department of Experimental Animal Science, Peking University Health Science Center, were placed in standard mouse cages for one week of acclimatization, during which normal feeding and water intake were ensured, and the indoor temperature and relative humidity were maintained at constant levels, with a 12 h light/dark cycle. Subsequently, the uric acid-lowering activity of three compounds were evaluated with a potassium oxonate-induced hyperuricemia mice model. The mice were randomly divided into control group, model group, allopurinol group, and nine groups treated with different concentrations of flavonoid compounds, with eight mice in each group. The model group and the flavonoid compound groups were administered potassium oxonate (300 mg/kg) via intraperitoneal injection to establish the hyperuricemic mice model. The control group received an intraperitoneal injection of an equal volume of 0.5% CMC-Na solution. One hour after modeling, the allopurinol group was administered allopurinol (5 mg/kg) via oral administration. The control group and model group were orally administered an equal volume of 0.5% CMC-Na solution. In the flavonoid compound treatment groups, the mice were orally administered a series of concentrations of engeletin (50, 100, and 200 mg/kg), neoisoastilbin (25, 50, and 100 mg/kg), and naringenin (25, 50, and 100 mg/kg). One hour after administration, the eyes of mice in each group were enucleated, and then blood was collected and left to stand at 4 °C for 1 h. Subsequently, the blood samples were centrifugated twice at a speed of 5000 rpm (2054× *g*) at 4 °C for 10 min. Then the supernatant was collected as serum, stored at −20 °C, and used for measuring serum-related indicators. The serum concentrations of uric acid (UA) were determined using a uric acid detection kit according to the manufacturer’s instructions.

### 3.3. Instruments and Conditions

UHPLC-Q-TOF-MS analysis was performed on the SCIEX Triple TOF 6600+ and SCIEX Exion LC AD System. All data were processed and analyzed using PeakView V.1.2 software. An ACQUITY UPLC BEH C18 column (2.1 × 150 mm, 1.7 μm, Waters, Milford, MA, USA) with a VanGuard Pre-Column (2.1 × 5 mm, 1.7 μm, Waters, USA) was used for chromatographic separation. The injection volume was 2 μL and the flow rate was 0.3 mL/min. The column temperature was set to 40 °C. The composition of the mobile phase was 0.1% formic acid (A) and acetonitrile (B). The gradient elution procedure was as follows: 0–5 min, 2% B; 5–20 min, 2–5% B; 20–40 min, 5–8% B; 40–60 min, 8–10% B; 60–75 min, 10–12% B; 75–85 min, 12–14% B; 85–88 min, 14% B; 88–100 min, 14–16% B; 100–120 min, 16% B; 120–125 min, 16–22% B; 125–135 min, 22–35% B; 135–145 min, 35–55% B; 145–148 min, 55–82% B; 148–150 min, 82–100% B; 150–152 min, 100–2% B; 152–160 min, 2% B. The MS parameters were as follows: negative ion detection with electrospray ionization (ESI); scan range, *m*/*z* 100–2000 (MS) and 50–2000 (MS^2^); collision energy, 35 ± 15 eV; Gas 1, 60 psi; Gas 2, 60 psi; curtain gas, 35 psi; ion source temperature, 600 °C; ion spray voltage, −4500 V; declustering potential, −60 V; collision energy, 35 ± 15 eV.

### 3.4. Identification of the Existence Forms of Engeletin in Mice Samples

The strategy previously reported by the authors [57] was employed to identify and characterize the “in vivo existence forms” of engeletin. First, the base peak chromatograms (BPCs) of the treated group samples were compared with those of the blank group samples to identify differential chromatographic peaks and preliminarily determine potential in vivo existence forms. Second, the extracted ion chromatograms (EICs) of the compounds in the treated group samples and the blank group samples were compared to confirm and validate the identified differences. Chromatographic peaks present in the treated group samples but not in the blank group samples were identified as the in vivo existence forms of engeletin. Finally, the structures of in vivo existence forms were tentatively elucidated based on the obtained UHPLC-Q-TOF-MS/MS data, combined with the MS data of the reference compounds and MS data reported in the literature by searching the SciFinder database.

### 3.5. Assay of Inhibitory Activity on Xanthine Oxidase In Vitro

The xanthine oxidase inhibition assay was carried out according to the previous method with slight modification [34]. First, 100 μL of various concentrations of samples, and 50 μL of xanthine oxidase solution (25 U/L) were added to a 96-well plate in sequence. Then the mixture was equilibrated at 37 °C for 30 min. Finally, 50 μL of substrate (0.5 mmol/L xanthine) was added to initiate the reaction. The absorbance was measured at a wavelength of 295 nm after 5 min. The resulting value of this measurement was recorded. Allopurinol was used as the positive control. PBS buffer was used as blank control. Each sample was prepared in triplicate and tested three times. The formula for calculating the inhibitory activity of xanthine oxidase is as follows:

$$Inhibit\;ratio\;(\%)=\frac{\left[{\left(\frac{d_{A}}{d_{t}}\right)}_{blank}-{\left(\frac{d_{A}}{d_{t}}\right)}_{sample}\right]}{{\left(\frac{d_{A}}{d_{t}}\right)}_{blank}}\times 100\%$$

where (*d_A_*/*d_t_*)_blank_ and (*d_A_*/*d_t_*)_sample_ denote the reaction rate of blank and sample group respectively.

### 3.6. Molecular Docking

The protein crystal structure of xanthine oxidase (PDB ID: 3nvy) [58] was obtained from the RCSB Protein Data Bank (PDB) database (https://www.rcsb.org/, accessed on 5 February 2025), with a resolution of 2.00 Å. The structural formulas of neoisoastilbin (PubChem CID: 10114809), naringenin (PubChem CID: 439246), and allopurinol (PubChem CID: 135401907) were retrieved from the PubChem database (https://pubchem.ncbi.nlm.nih.gov/, accessed on 8 March 2025), and their 3D structural information in SDF format was downloaded. All small molecules and water molecules from the obtained xanthine oxidase protein structure were removed, and then the structure files of the protein and the three compounds were converted into pdbqt files using MGLTools 1.5.7 software. Subsequently, a docking box was constructed with its center at x = 39, y = 21, z = 20 and dimensions of 40 Å × 40 Å × 40 Å, with a grid spacing of 0.375 Å. Then, Autodock vina 1.1.2 software was used to dock the small molecule with the protein; the docking mode was semi-flexible, and the docking algorithm was the Lamarckian genetic algorithm. Finally, the docking results of the receptor and ligand were visualized by using PyMOL 2.3.0 and Discovery Studio 2019. Binding energies are presented as the mean ± standard deviation (mean ± SD) from three independent docking calculations.

### 3.7. Statistical Analysis

The data of inhibitory activity of the compounds on xanthine oxidase are expressed as mean ± standard deviation (mean ± SD). The data of uric acid-lowering activity of the compounds in vivo are expressed as the mean ± standard error of mean (mean ± SEM). All statistical analyses were conducted with GraphPad Prism 10.1.2 software (San Diego, CA, USA). One-way analysis of variance (ANOVA) was performed to determine the significant differences, and *p* < 0.05 was considered statistically significant.

## 4. Conclusions

In summary, engeletin is a dihydroflavonol glycoside compound found in Smilacis Glabrae Rhizoma, and there are currently no published reports on its metabolism. This study demonstrates the in vivo uric acid-lowering effect of engeletin and its metabolic process in mice for the first time. A total of 11, 34, 7, 6, and 5 compounds were detected in urine, feces, serum, liver, and kidney samples from mice administered with engeletin, respectively. Subsequent to the elimination of duplicate compounds, a total of 52 compounds were identified, inclusive of the parent compound engeletin and its 51 metabolites. It was evident that the structures of the metabolites provided a foundation for the inference of the metabolic pathways of engeletin in mice. The pathways involved a multitude of metabolic reactions, including sulfation, glucuronidation, hydroxylation, methylation, and hydrolysis. The predominant metabolic reactions are glucuronidation, sulfation, and hydrolysis, which accounted for 15, 13, and 11 metabolites, respectively. The metabolites of engeletin contain 16 phase I metabolic products and 35 phase II metabolic products. All the 51 metabolites are newly discovered metabolites of engeletin, among which 16 are potential new compounds. This study was the first to demonstrate that engeletin could be converted into other flavonoid constituents of Smilacis Glabrae Rhizoma in mice, namely neoisoastilbin (E28), dihydrokaempferol (E40), taxifolin (E48) and naringenin (E50). These observations indicated that engeletin might exert its effects by converting into other flavonoid compounds in vivo. Subsequently, an investigation was conducted into the in vitro and/or in vivo activities of engeletin and its four flavonoid metabolites. The results demonstrated that, despite the absence of xanthine oxidase inhibitory activity in vitro, engeletin exhibited uric acid-lowering effects in vivo. Among the four metabolites of engeletin, neoisoastilbin and naringenin showed both in vitro xanthine oxidase inhibitory activity and in vivo uric acid-lowering effects. These findings suggested that the effective forms responsible for the ability of engeletin to reduce serum uric acid levels in mice with hyperuricemia might be part of its metabolites. This study established a foundation for understanding the in vivo metabolism of dihydroflavonol glycoside compounds, and also elucidated the potential mechanism underlying the uric acid-lowering effect of engeletin on hyperuricemia. Building upon the metabolic conversion from the parent compound to its metabolites, the precise mechanism by which the metabolites of engeletin exert their uric acid-lowering effects in vivo can be further validated and explored. Furthermore, engeletin is a flavonoid glycoside, and its in vivo activity after oral administration is likely influenced by its pharmacokinetic behavior, such as intestinal metabolism and gut microbiota-mediated transformation. In the gastrointestinal tract, engeletin may be hydrolyzed to release the corresponding aglycone, which can subsequently undergo microbial biotransformation and phase II metabolism, such as glucuronidation and sulfation. However, it should be noted that the metabolic fate of engeletin may differ between mice and humans because of interspecies differences in drug-metabolizing enzyme profiles, as well as differences in intestinal bacterial colonization and microbiota composition [59,60]. Such differences may change the types of metabolites of engeletin and thereby influence efficacy and exposure in humans. Therefore, further pharmacokinetic and microbiota-related studies are required before extrapolating these findings to human hyperuricemia.

## Figures and Tables

**Table 1:** Engeletin and its 51 new metabolites in mice urine, feces, serum, liver and kidney samples.

No.	t_R_ (min)	Formula	Ion	Meas. *m*/*z*	Pred. *m*/*z*	Diff (ppm)	Identification	U	F	S	L	K
E0 ^a^	82.29	C_21_H_22_O_10_	[M−H]^−^	433.1135	433.1140	−1.2	engeletin	−	+	+	−	−
E1	80.88	C_21_H_22_O_10_	[M−H]^−^	433.1130	433.1140	−2.4	engeletin isomer	−	+	−	+	+
E2	83.80	C_21_H_22_O_10_	[M−H]^−^	433.1151	433.1140	2.5	engeletin isomer	+	−	−	−	−
E3	92.52	C_21_H_22_O_10_	[M−H]^−^	433.1133	433.1140	−1.7	engeletin isomer	−	+	−	−	−
E4	98.06	C_21_H_22_O_10_	[M−H]^−^	433.1140	433.1140	0.0	engeletin isomer	−	−	−	+	−
E5	100.18	C_21_H_22_O_10_	[M−H]^−^	433.1138	433.1140	−0.5	engeletin isomer	−	+	−	−	−
E6	25.58	C_21_H_22_O_13_S	[M−H]^−^	513.0705	513.0708	−0.7	engeletin sulfate or isomer	−	+	−	−	−
E7	56.58	C_21_H_22_O_13_S	[M−H]^−^	513.0695	513.0708	−2.6	engeletin sulfate or isomer	−	+	−	−	−
E8	58.32	C_21_H_22_O_13_S	[M−H]^−^	513.0693	513.0708	−3.0	engeletin sulfate or isomer	−	+	−	−	−
E9	68.85	C_21_H_22_O_13_S	[M−H]^−^	513.0700	513.0708	−1.6	engeletin sulfate or isomer	−	+	−	−	−
E10	70.19	C_21_H_22_O_13_S	[M−H]^−^	513.0691	513.0708	−3.4	engeletin sulfate or isomer	+	−	−	−	−
E11 ^n^	31.75	C_27_H_30_O_16_	[M−H]^−^	609.1472	609.1461	1.8	engeletin glucuronide or isomer	−	−	+	−	−
E12 ^n^	33.02	C_27_H_30_O_16_	[M−H]^−^	609.1465	609.1461	0.6	engeletin glucuronide or isomer	−	−	−	+	+
E13 ^n^	34.91	C_27_H_30_O_16_	[M−H]^−^	609.1471	609.1461	1.6	engeletin glucuronide or isomer	+	−	+	−	−
E14 ^n^	44.31	C_27_H_30_O_16_	[M−H]^−^	609.1456	609.1461	−0.8	engeletin glucuronide or isomer	−	−	−	+	+
E15 ^n^	45.76	C_27_H_30_O_16_	[M−H]^−^	609.1460	609.1461	−0.2	engeletin glucuronide or isomer	−	−	+	−	−
E16 ^n^	49.85	C_27_H_30_O_16_	[M−H]^−^	609.1473	609.1461	2.0	engeletin glucuronide or isomer	+	−	−	−	−
E17 ^n^	52.19	C_27_H_30_O_16_	[M−H]^−^	609.1471	609.1461	1.6	engeletin glucuronide or isomer	+	−	+	−	−
E18 ^n^	54.81	C_27_H_30_O_16_	[M−H]^−^	609.1451	609.1461	−1.7	engeletin glucuronide or isomer	−	−	−	+	+
E19 ^n^	58.06	C_27_H_30_O_16_	[M−H]^−^	609.1479	609.1461	2.9	engeletin glucuronide or isomer	+	−	+	−	−
E20 ^n^	60.46	C_27_H_30_O_16_	[M−H]^−^	609.1450	609.1461	−1.8	engeletin glucuronide or isomer	−	−	−	+	+
E21 ^n^	63.55	C_27_H_30_O_16_	[M−H]^−^	609.1473	609.1461	2.0	engeletin glucuronide or isomer	+	−	+	−	−
E22 ^n^	76.93	C_27_H_30_O_16_	[M−H]^−^	609.1444	609.1461	−2.8	engeletin glucuronide or isomer	−	+	−	−	−
E23	40.95	C_27_H_32_O_16_	[M−H]^−^	611.1640	611.1618	3.7	dihydro-engeletin glucuronide or isomer	−	+	−	−	−
E24	45.08	C_27_H_32_O_16_	[M−H]^−^	611.1633	611.1618	2.5	dihydro-engeletin glucuronide or isomer	−	+	−	−	−
E25	59.74	C_27_H_32_O_16_	[M−H]^−^	611.1632	611.1618	2.4	dihydro-engeletin glucuronide or isomer	−	+	−	−	−
E26	64.46	C_21_H_22_O_11_	[M−H]^−^	449.1077	449.1089	−2.8	hydroxy-engeletin or isomer	−	+	−	−	−
E27	67.02.	C_21_H_22_O_11_	[M−H]^−^	449.1082	449.1089	−1.6	hydroxy-engeletin or isomer	−	+	−	−	−
E28 ^a^	79.64	C_21_H_22_O_11_	[M−H]^−^	449.1071	449.1089	−4.1	neoisoastilbin	−	+	−	−	−
E29 ^n^	102.45	C_22_H_24_O_10_	[M−H]^−^	447.1285	447.1297	−2.6	methyl-engeletin	−	+	−	−	−
E30 ^n^	53.22	C_22_H_24_O_13_S	[M−H]^−^	527.0851	527.0865	−2.6	methyl-engeletin sulfate or isomer	−	+	−	−	−
E31 ^n^	68.20	C_22_H_24_O_13_S	[M−H]^−^	527.0845	527.0865	−3.8	methyl-engeletin sulfate or isomer	−	+	−	−	−
E32	71.50	C_22_H_24_O_11_	[M−H]^−^	463.1231	463.1246	−3.2	methyl-hydroxy-engeletin or isomer	−	+	−	−	−
E33	86.31	C_22_H_24_O_11_	[M−H]^−^	463.1228	463.1246	−3.9	methyl-hydroxy-engeletin or isomer	−	+	−	−	−
E34	97.25	C_22_H_24_O_11_	[M−H]^−^	463.1235	463.1246	−2.3	methyl-hydroxy-engeletin or isomer	−	+	−	−	−
E35	87.09	C_22_H_24_O_11_	[M−H]^−^	463.1260	463.1246	3.1	methyl-hydroxy-engeletin or isomer	+	−	−	−	−
E36	48.42	C_20_H_20_O_10_	[M−H]^−^	419.0976	419.0984	−1.8	demethyl-engeletin	−	+	−	−	−
E37	55.19	C_22_H_22_O_10_	[M−H]^−^	445.1124	445.1140	−3.6	methyl-dehydrogenated-engeletin or isomer	−	+	−	−	−
E38	54.49	C_22_H_22_O_10_	[M−H]^−^	445.1138	445.1140	−0.5	methyl-dehydrogenated-engeletin or isomer	−	+	−	−	−
E39 ^n^	103.77	C_23_H_26_O_11_	[M−H]^−^	477.1397	477.1402	−1.1	acetylated-hydrogenated-engeletin	−	+	−	−	−
E40 ^a^	67.35	C_15_H_12_O_6_	[M−H]^−^	287.0567	287.0561	2.0	dihydrokaempferol	−	+	−	−	−
E41	68.23	C_15_H_12_O_6_	[M−H]^−^	287.0564	287.0561	1.0	dihydrokaempferol isomer	+	−	−	−	−
E42	33.79	C_15_H_12_O_9_S	[M−H]^−^	367.0126	367.0129	−0.9	dihydrokaempferol sulfate or isomer	+	−	−	−	−
E43	38.42	C_15_H_12_O_9_S	[M−H]^−^	367.0124	367.0129	−1.4	dihydrokaempferol sulfate or isomer	+	−	−	−	−
E44	42.08	C_15_H_12_O_9_S	[M−H]^−^	367.0121	367.0129	−2.3	dihydrokaempferol sulfate or isomer	−	+	−	−	−
E45	50.43	C_15_H_12_O_9_S	[M−H]^−^	367.0120	367.0129	−2.5	dihydrokaempferol sulfate or isomer	−	+	−	−	−
E46	49.03	C_15_H_14_O_9_S	[M−H]^−^	369.0269	369.0286	−4.5	dihydro-dihydrokaempferol sulfate	−	+	−	−	−
E47	67.06	C_21_H_22_O_11_	[M−H]^−^	449.1082	449.1089	−1.6	dihydrokaempferol glucoside	−	+	−	−	−
E48 ^a^	48.44	C_15_H_12_O_7_	[M−H]^−^	303.0505	303.0510	−1.7	taxifolin	−	+	−	−	−
E49	78.02	C_15_H_12_O_5_	[M−H]^−^	271.0604	271.0612	−2.9	naringenin isomer	−	+	−	−	−
E50 ^a^	123.55	C_15_H_12_O_5_	[M−H]^−^	271.0604	271.0612	−2.9	naringenin	−	+	−	−	−
E51	40.16	C_16_H_14_O_9_S	[M−H]^−^	381.0271	381.0286	−3.9	methyl-dihydrokaempferol sulfate	−	+	−	−	−

t_R_: retention time; Meas.: measured; Pred.: predicted; Diff: difference; U: urine; F: feces; S: serum; L: liver; K: kidney; +: detected; −: undetected; ^n^: new compound; ^a^: comparison with reference compounds.

**Table 2:** Formative metabolic reactions of engeletin metabolites in mice.

Metabolite	Number	Metabolic Reaction											
		Phase I Metabolism							Phase II Metabolism				
		−OH	+OH	−2H	+2H	−CH_3_	Hyds	Iso	+CH_3_	+SO_3_H	+GlcUA	+Glc	+Acy
E1–E5	5							●					
E6–E10	5									●			
E11–E22	12										●		
E23–E25	3				●						●		
E26–E28	3		●										
E29	1								●				
E30–E31	2								●	●			
E32–E35	4		●						●				
E36	1					●							
E37–E38	2			●					●				
E39	1												●
E40–E41	2						●						
E42–E45	4						●			●			
E46	1				●		●			●			
E47	1						●					●	
E48	1		●				●						
E49–E50	2	●					●						
E51	1								●	●			
Sum	51	2	8	2	4	1	11	5	10	13	15	1	1

−OH, dehydroxylation; +OH, hydroxylation; −2H, dehydrogenation; +2H, hydrogenation; +CH_3_, methylation; Hyds, hydrolysis; Iso, isomerization; +SO_3_, sulfation; +GlcUA, glucuronidation; +Glc, glycosylation; Acy, acetylation; ●, possessing this type of metabolic reaction.

## Data Availability

The data presented in this study are contained within the article and its Supplementary Materials. Further inquiries can be directed to the corresponding authors.

## Supplementary Materials

The following supporting information can be downloaded at https://www.mdpi.com/article/10.3390/ijms27125353/s1.
