# Supplementary material for: The In Vivo Existence Forms of Engeletin and Their Anti-Hyperuricemia Activity
Source: Int J Mol Sci. 2026 Jun 13;27(12):5353. doi: 10.3390/ijms27125353 (PMC13299705; doi:10.3390/ijms27125353)
Supplement: Supplementary file 1 [file ijms-27-05353-s001.zip › ijms-4342089-supplementary.pdf]

Supplementary Material

1. Table S1. UHPLC-Q-TOF-MS/MS data of the metabolites of engeletin in mice under negative ion mode

2. Table S2. Molecular docking results of two active metabolites and allopurinol with xanthine oxidase

3. Figure S1. The Base Peak Chromatograms (BPCs) of drug-containing samples of mice urine in negative and positive ion modes

4. Figure S2. The BPCs of samples of mice urine and feces

5. Figure S3. The BPCs of samples of mice serum and liver

6. Figure S4. The BPCs of samples of mice kidney

7. Figure S5. The extracted ion chromatograms (EICs) of compounds (E2, E10, E13, E16, E17, E19, E21, E35, E41–E43) in mice urine after administration of engeletin

8. Figure S6. The EICs of compounds (E7–E9, E22, E25–E27, E32, E40, E47) in mice feces after administration of engeletin

9. Figure S7. The EICs of compounds (E0, E1, E3, E5, E28, E29, E31, E33, E34, E39, E49, E50) in mice feces after administration of engeletin

10. Figure S8. The EICs of compounds (E6, E23, E24, E30, E36–E38, E44–E46, E48, E51) in mice feces after administration of engeletin

11. Figure S9. The EICs of compounds (E0, E11, E13, E15, E17, E19, E21) in mice serum after administration of engeletin

12. Figure S10. The EICs of compounds (E1, E4, E12, E14, E18, E20) in mice liver after administration of engeletin

13. Figure S11. The EICs of compounds (E1, E12, E14, E18, E20) in mice kidney after administration of engeletin

14. Figure S12. Inhibition rate of engeletin, dihydrokaempferol and taxifolin on xanthine oxidase

Table S1. UHPLC-Q-TOF-MS/MS data of the metabolites of engeletin in mice under negative ion mode

No.	t_R (min)	Formula	Meas. *m/z*	Diff (ppm)	Identification	MS/MS fragment ions
E0	82.29	$C_{21}H_{22}O_{10}$	433.1135	−1.2	engeletin	269.0456, 259.0622, 180.0067, 152.0120
E1	80.88	$C_{21}H_{22}O_{10}$	433.1130	−2.4	engeletin isomer	287.0611, 269.0441, 259.0605, 180.0060, 152.0117
E2	83.80	$C_{21}H_{22}O_{10}$	433.1151	2.5	engeletin isomer	287.0576, 269.0475, 259.0531, 180.0080, 152.0118
E3	92.52	$C_{21}H_{22}O_{10}$	433.1133	−1.7	engeletin isomer	287.0561, 269.0455, 259.0616, 180.0029, 152.0119
E4	98.06	$C_{21}H_{22}O_{10}$	433.1140	0.0	engeletin isomer	180.0062, 152.0109, 125.0279
E5	100.18	$C_{21}H_{22}O_{10}$	433.1138	−0.5	engeletin isomer	287.0554, 269.0437, 259.0631, 180.0069, 152.0103
E6	25.58	$C_{21}H_{22}O_{13}S$	513.0705	−0.7	engeletin sulfate or isomer	431.0991, 284.0316, 255.0287, 227.0331
E7	56.58	$C_{21}H_{22}O_{13}S$	513.0695	−2.6	engeletin sulfate or isomer	431.0984
E8	58.32	$C_{21}H_{22}O_{13}S$	513.0693	−3.0	engeletin sulfate or isomer	433.1103, 269.0452, 180.0048, 152.0120
E9	68.85	$C_{21}H_{22}O_{13}S$	513.0700	−1.6	engeletin sulfate or isomer	433.1166, 287.0572, 269.0458, 259.0609, 243.0654, 180.0072, 152.0098
E10	70.19	$C_{21}H_{22}O_{13}S$	513.0691	−3.4	engeletin sulfate or isomer	433.1160, 367.0162, 287.0578, 269.0482, 259.0629, 180.0059, 152.0108
E11	31.75	$C_{27}H_{30}O_{16}$	609.1472	1.8	engeletin glucuronide or isomer	433.1148, 287.0585, 269.0474, 259.0582, 243.0662, 180.0038, 152.0063

No.	t_R (min)	Formula	Meas. *m/z*	Diff (ppm)	Identification	MS/MS fragment ions
E12	33.02	$C_{27}H_{30}O_{16}$	609.1465	0.6	engeletin glucuronide or isomer	433.1170, 359.0771, 329.0714, 301.0698, 287.0573, 269.0444, 259.0601, 243.0666, 180.0061, 152.0133
E13	34.91	$C_{27}H_{30}O_{16}$	609.1471	1.6	engeletin glucuronide or isomer	433.1162, 287.0569, 269.0471, 259.0583, 243.0664, 180.0059, 152.0084
E14	44.31	$C_{27}H_{30}O_{16}$	609.1456	−0.8	engeletin glucuronide or isomer	433.1161, 329.0652, 287.0576, 269.0470, 259.0610
E15	45.76	$C_{27}H_{30}O_{16}$	609.1460	−0.2	engeletin glucuronide or isomer	433.1147, 287.0578, 269.0446, 259.0273, 243.0689, 180.0069, 152.0077
E16	49.85	$C_{27}H_{30}O_{16}$	609.1473	2.0	engeletin glucuronide or isomer	433.1165, 287.0568, 269.0466, 259.0624, 151.0050
E17	52.19	$C_{27}H_{30}O_{16}$	609.1471	1.6	engeletin glucuronide or isomer	433.1170, 287.0574, 269.0472, 259.0623, 179.0001, 151.0033
E18	54.81	$C_{27}H_{30}O_{16}$	609.1451	−1.7	engeletin glucuronide or isomer	433.1067, 287.0610, 269.0501, 259.0273, 243.0689, 180.0069, 152.0114
E19	58.06	$C_{27}H_{30}O_{16}$	609.1479	2.9	engeletin glucuronide or isomer	433.1145, 287.0555, 269.0434, 259.0598, 243.0718, 178.9997, 151.0069
E20	60.46	$C_{27}H_{30}O_{16}$	609.1450	−1.8	engeletin glucuronide or isomer	433.1186, 287.0551, 269.0437, 180.0098
E21	63.55	$C_{27}H_{30}O_{16}$	609.1473	2.0	engeletin glucuronide or isomer	433.1169, 287.0576, 269.0461, 259.0634, 243.0682, 178.9979, 151.0045
E22	76.93	$C_{27}H_{30}O_{16}$	609.1444	−2.8	engeletin glucuronide or isomer	433.1163, 287.0599, 269.0481, 259.0606, 243.0756

No.	t_R (min)	Formula	Meas. *m/z*	Diff (ppm)	Identification	MS/MS fragment ions
E23	40.95	$C_{27}H_{32}O_{16}$	611.1640	3.7	dihydro-engeletin glucuronide or isomer	523.1522, 435.1350, 405.1269, 303.1135
E24	45.08	$C_{27}H_{32}O_{16}$	611.1633	2.5	dihydro-engeletin glucuronide or isomer	523.1523, 505.1372, 479.1632, 435.1384, 405.1220, 329.1229, 227.1192, 215.0850
E25	59.74	$C_{27}H_{32}O_{16}$	611.1632	2.4	dihydro-engeletin glucuronide or isomer	435.1444, 405.1247, 327.1318, 215.0874
E26	64.46	$C_{21}H_{22}O_{11}$	449.1077	−2.8	hydroxy-engeletin or isomer	269.0414, 259.0656
E27	67.02	$C_{21}H_{22}O_{11}$	449.1082	−1.6	hydroxy-engeletin or isomer	303.0506, 285.0398, 151.0041
E28	79.64	$C_{21}H_{22}O_{11}$	449.1071	−4.1	neoisoastilbin	285.0397, 151.0028
E29	102.45	$C_{22}H_{24}O_{10}$	447.1285	−2.6	methyl-engeletin	269.0448, 180.0063, 152.0104
E30	53.22	$C_{22}H_{24}O_{13}S$	527.0851	−2.6	methyl-engeletin sulfate or isomer	445.1152, 281.0468, 163.0052
E31	68.20	$C_{22}H_{24}O_{13}S$	527.0845	−3.8	methyl-engeletin sulfate or isomer	445.1096, 163.0061
E32	71.50	$C_{22}H_{24}O_{11}$	463.1231	−3.2	methyl-hydroxy-engeletin or isomer	445.1129, 281.0451, 163.0050
E33	86.31	$C_{22}H_{24}O_{11}$	463.1228	−3.9	methyl-hydroxy-engeletin or isomer	289.0729, 180.0049, 152.0096
E34	97.25	$C_{22}H_{24}O_{11}$	463.1235	−2.3	methyl-hydroxy-engeletin or isomer	299.0602, 289.0701, 180.0068, 152.0122
E35	87.09	$C_{22}H_{24}O_{11}$	463.1260	3.1	methyl-hydroxy-engeletin or isomer	180.0078, 152.0124
E36	48.42	$C_{20}H_{20}O_{10}$	419.0976	−1.8	demethyl-engeletin	401.0869, 359.0804, 341.0671, 329.0694

No.	t_R (min)	Formula	Meas. *m/z*	Diff (ppm)	Identification	MS/MS fragment ions
E37	55.19	$C_{22}H_{22}O_{10}$	445.1124	−3.6	methyl-dehydrogenated-engeletin or isomer	299.0599, 281.0480, 163.0037, 135.0474
E38	54.49	$C_{22}H_{22}O_{10}$	445.1138	−0.5	methyl-dehydrogenated-engeletin or isomer	281.0479, 248.1010, 220.1117, 204.1140, 186.1277, 164.0112, 137.0229
E39	103.77	$C_{23}H_{26}O_{11}$	477.1397	−1.1	acetylated-hydrogenated-engeletin	445.1156, 281.0447, 163.0033
E40	67.35	$C_{15}H_{12}O_6$	287.0567	2.0	dihydrokaempferol	259.0614, 243.0678, 201.0553, 173.0612, 151.0036, 125.0249, 107.0144
E41	68.23	$C_{15}H_{12}O_6$	287.0564	1.0	dihydrokaempferol isomer	259.0620, 201.0635, 177.0588, 173.0660, 157.0659, 151.0060, 125.0254
E42	33.79	$C_{15}H_{12}O_9S$	367.0126	−0.9	dihydrokaempferol sulfate or isomer	287.0550, 259.0614, 165.0190, 121.0287
E43	38.42	$C_{15}H_{12}O_9S$	367.0124	−1.4	dihydrokaempferol sulfate or isomer	287.0557, 259.0627, 243.0677, 201.0564, 173.0594, 151.0071, 125.0253
E44	42.08	$C_{15}H_{12}O_9S$	367.0121	−2.3	dihydrokaempferol sulfate or isomer	287.0548, 269.0462, 232.9816, 203.9720
E45	50.43	$C_{15}H_{12}O_9S$	367.0120	−2.5	dihydrokaempferol sulfate or isomer	287.0553, 259.0608, 180.0046, 152.0088
E46	49.03	$C_{15}H_{14}O_9S$	369.0269	−4.5	dihydro-dihydrokaempferol sulfate	289.0713, 181.0212, 151.0014
E47	67.06	$C_{21}H_{22}O_{11}$	449.1082	−1.6	dihydrokaempferol glucoside	285.0375, 217.0493, 166.0747, 151.0043
E48	48.44	$C_{15}H_{12}O_7$	303.0505	−1.7	taxifolin	151.0046, 125.0247, 107.0117

No.	t_R (min)	Formula	Meas. *m/z*	Diff (ppm)	Identification	MS/MS fragment ions
E49	78.02	$C_{15}H_{12}O_5$	271.0604	−2.9	naringenin isomer	165.0197, 137.0219, 116.9950, 93.0341
E50	123.55	$C_{15}H_{12}O_5$	271.0604	−2.9	naringenin	161.0604, 151.0040, 119.0511, 107.0170
E51	40.16	$C_{16}H_{14}O_9S$	381.0271	−3.9	methyl-dihydrokaempferol sulfate	299.0555, 281.0467, 271.0644, 253.0503, 192.0036, 177.0219, 163.0023, 137.0251

t_R: retention time; Meas.: measured; Diff: difference

Table S2. Molecular docking results of two active metabolites and allopurinol with xanthine oxidase (*n* =3, mean ± SD)

No.	Compound	Binding Energy （kcal/mol）	Function Type	Amino Acid Binding Site
1	allopurinol	−6.27 ± 0.06	Conventional Hydrogen Bond	ALA1079, GLU802, THR1010
			π–π	PHE1009, PHE914
			π–Alkyl	ALA1078, ALA1079
2	neoisoastilbin	−6.90 ± 0.10	Conventional Hydrogen Bond	SER876, LYS771
			Carbon Hydrogen Bond	LYS771
			π–π	PHE649
			π–Alkyl	PHE1013, LEU648, LEU1014, PRO1076, VAL1011, LYS771
3	naringenin	−7.93 ± 0.06	Conventional Hydrogen Bond	ASN768, LEU873
			Carbon Hydrogen Bond	LEU873
			π–π	PHE649
			π–Alkyl	PRO1076, LEU648, VAL1011, LEU1014

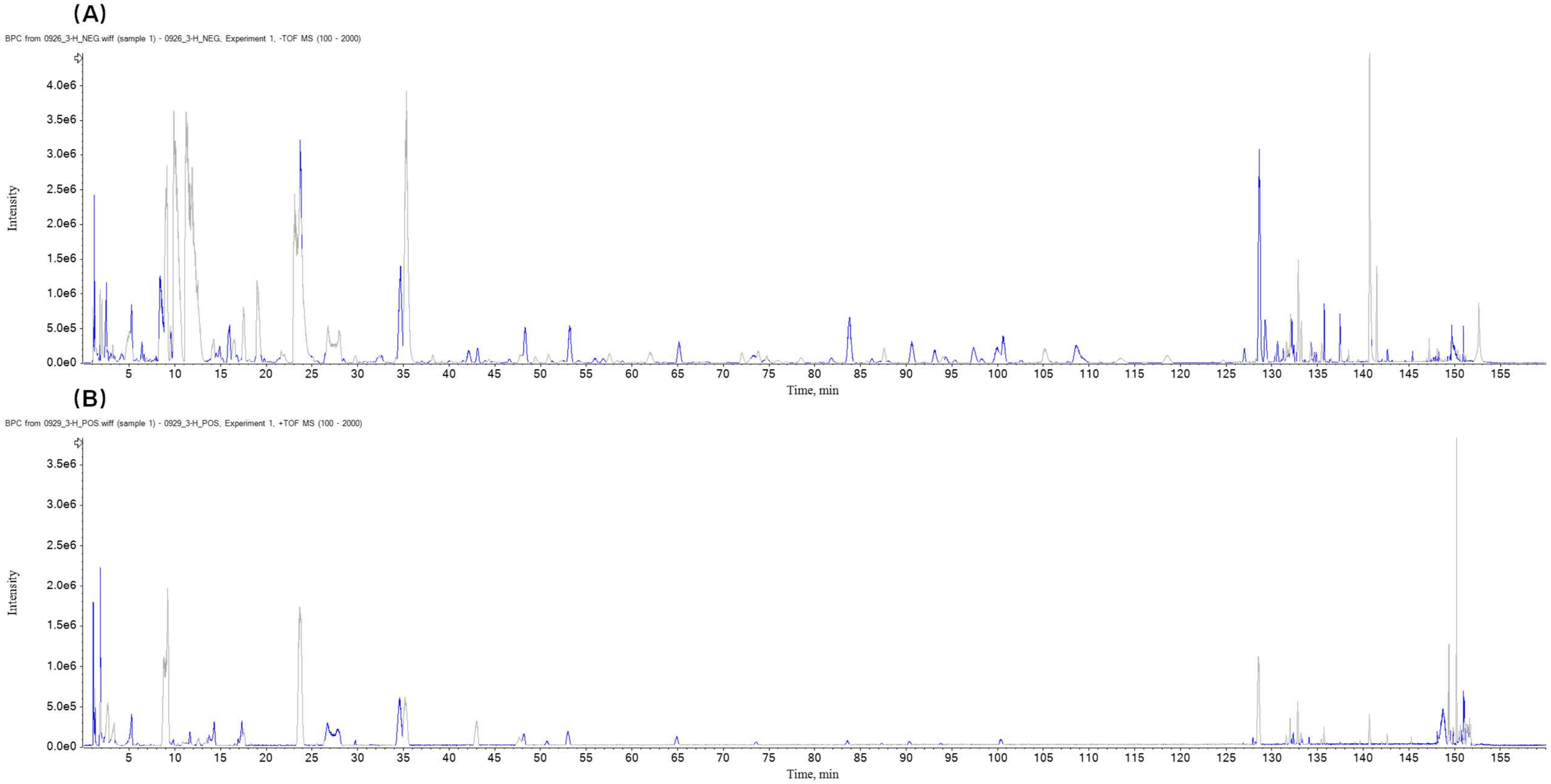

Figure S1. The Base Peak Chromatograms (BPCs) of drug-containing samples of mice urine in negative and positive ion modes. (A) Drug-containing sample of urine in negative ion mode; (B) Drug-containing sample of urine in positive ion mode. Blue lines represent the ion with the strongest mass spectrometric signal at each time point. Grey lines indicate points at which the *m/z* value of the strongest ion changes beyond the preset mass tolerance between adjacent scans, suggesting that a new major component with a different mass may be present at those points.

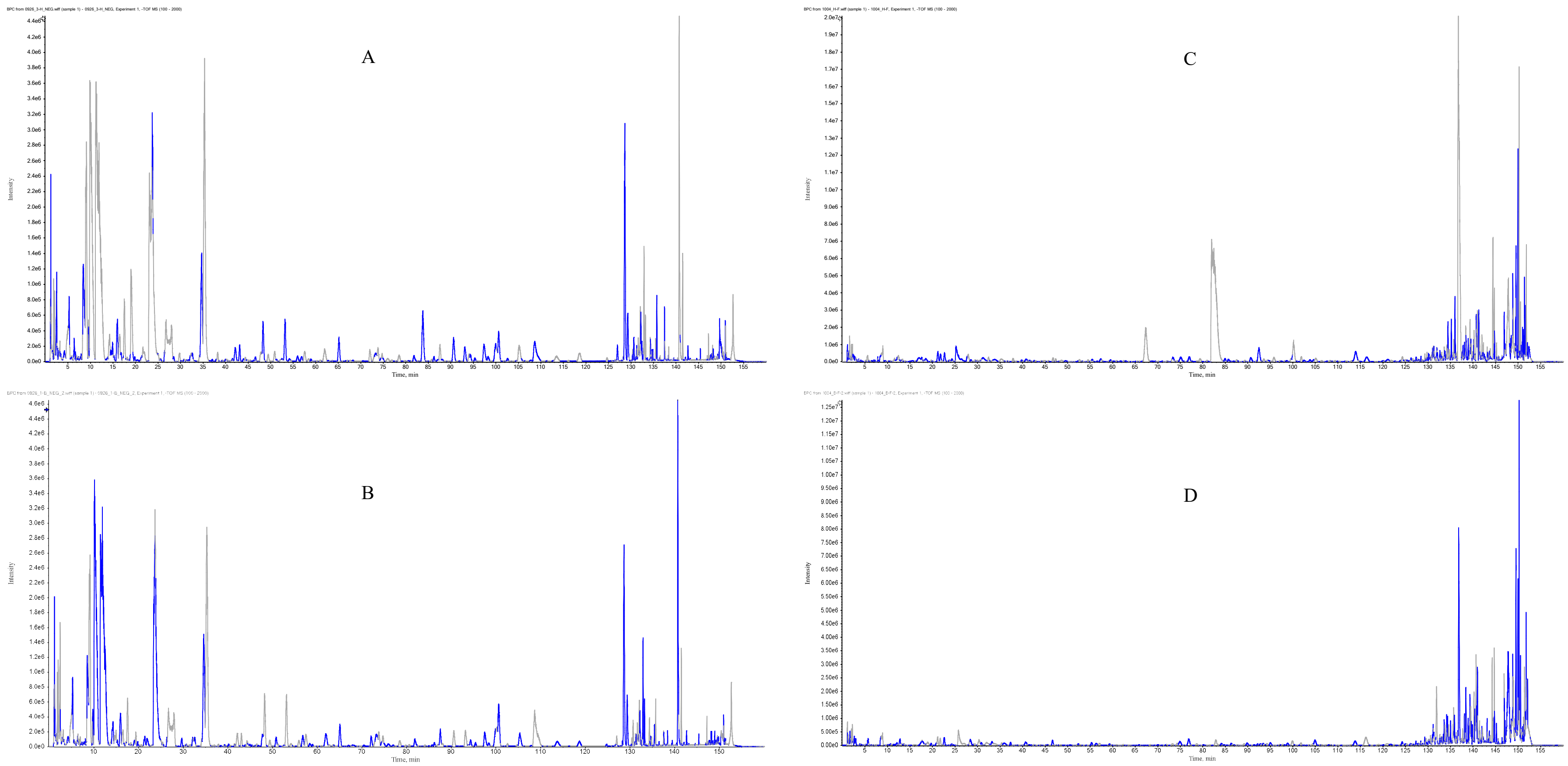

Figure S2. The BPCs of samples of mice urine and feces. (A) Drug-containing sample of urine. (B) Blank sample of urine. (C) Drug-containing sample of feces. (D) Blank sample of feces. Blue lines represent the ion with the strongest mass spectrometric signal at each time point. Grey lines indicate points at which the *m/z* value of the strongest ion changes beyond the preset mass tolerance between adjacent scans, suggesting that a new major component with a different mass may be present at those points.

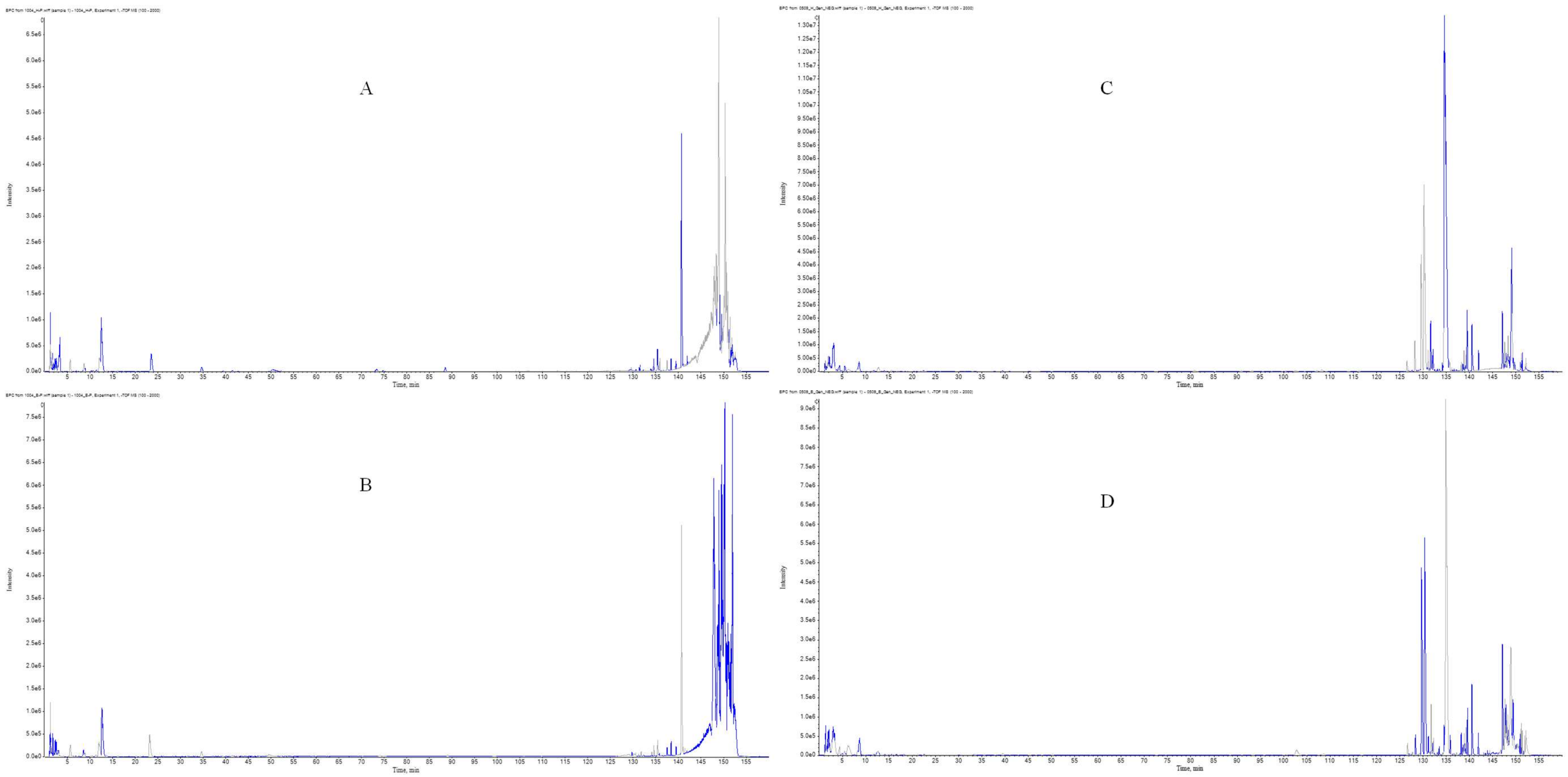

Figure S3. The BPCs of samples of mice serum and liver. (A) Drug-containing sample of serum. (B) Blank sample of serum. (C) Drug-containing sample of liver. (D) Blank sample of liver. Blue lines represent the ion with the strongest mass spectrometric signal at each time point. Grey lines indicate points at which the *m/z* value of the strongest ion changes beyond the preset mass tolerance between adjacent scans, suggesting that a new major component with a different mass may be present at those points.

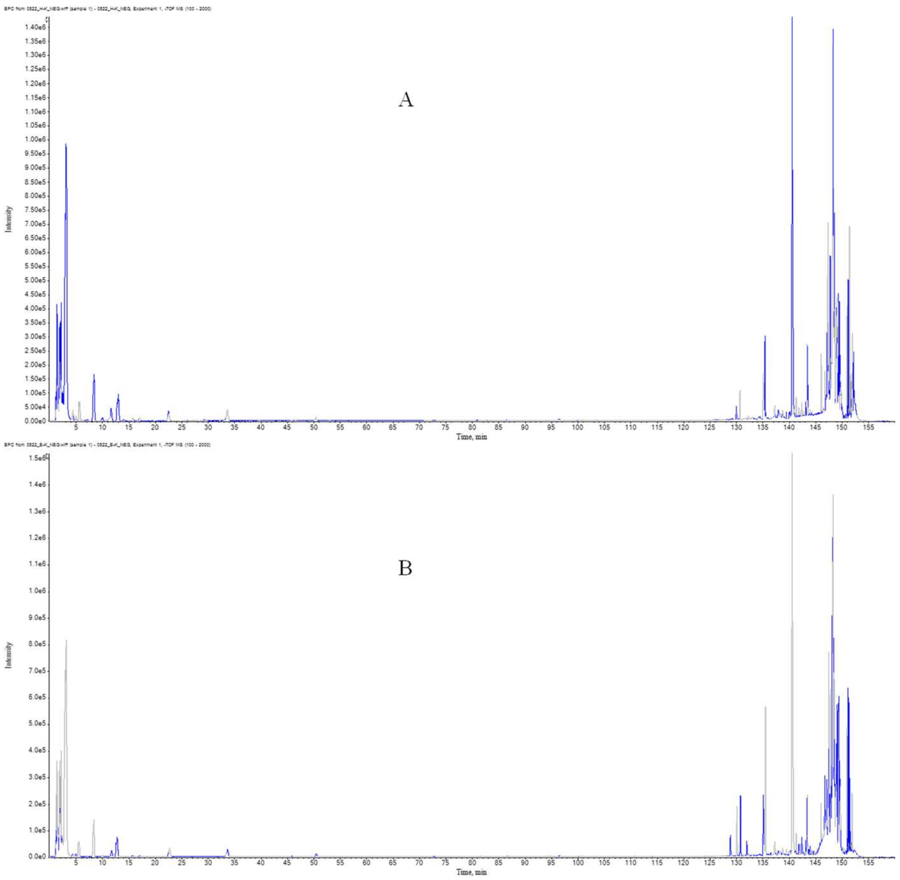

Figure S4. The BPCs of samples of mice kidney. (A) Drug-containing sample of kidney. (B) Blank sample of kidney. Blue lines represent the ion with the strongest mass spectrometric signal at each time point. Grey lines indicate points at which the *m/z* value of the strongest ion changes beyond the preset mass tolerance between adjacent scans, suggesting that a new major component with a different mass may be present at those points.

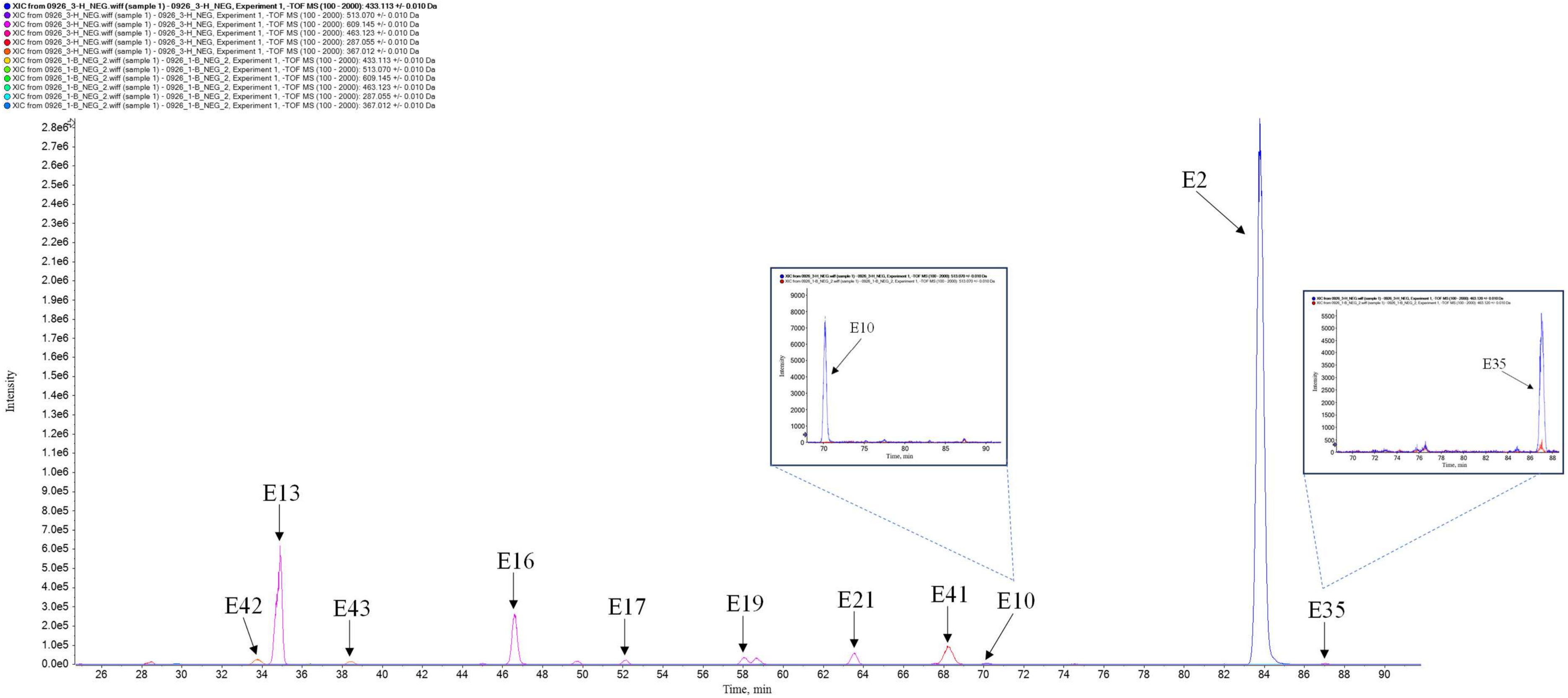

Figure S5. The extracted ion chromatograms (EICs) of 11 compounds (E2, E10, E13, E16, E17, E19, E21, E35, E41–M43) in mice urine after administration of engeletin

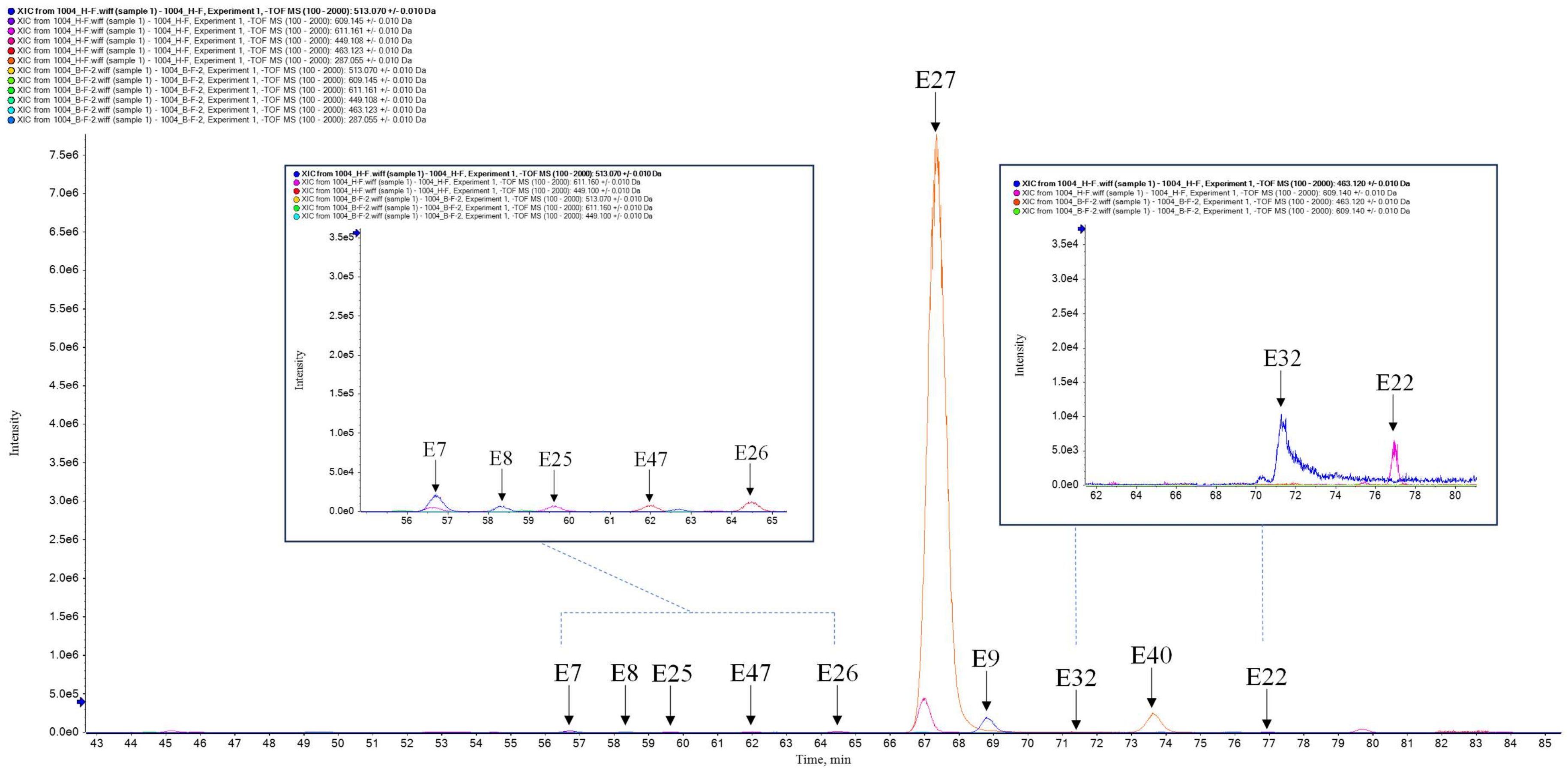

Figure S6. The EICs of compounds (E7–E9, E22, E25–E27, E32, E40, E47) in mice feces after administration of engeletin

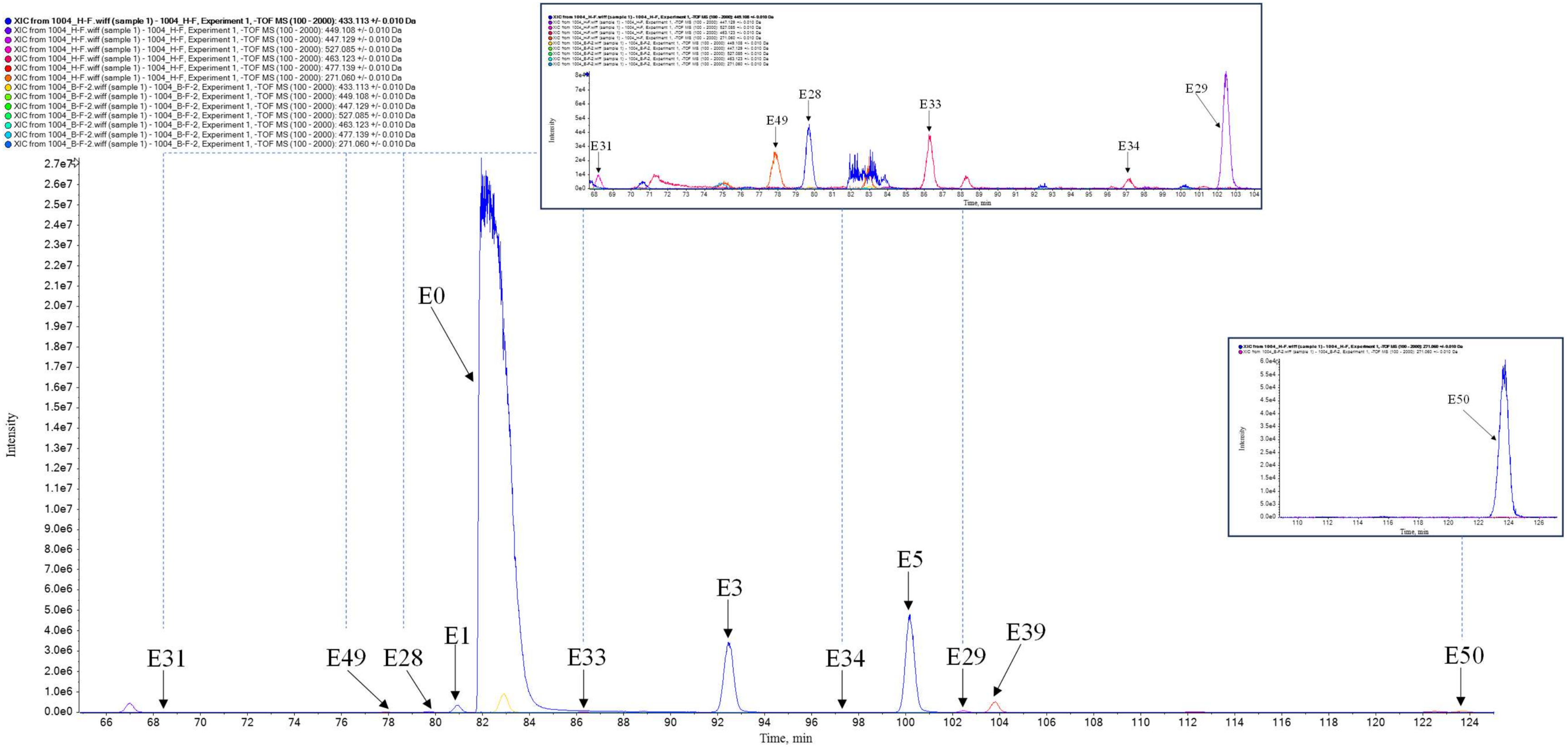

Figure S7. The EICs of compounds (E0, E1, E3, E5, E28, E29, E31, E33, E34, E39, E49, E50) in mice feces after administration of engeletin

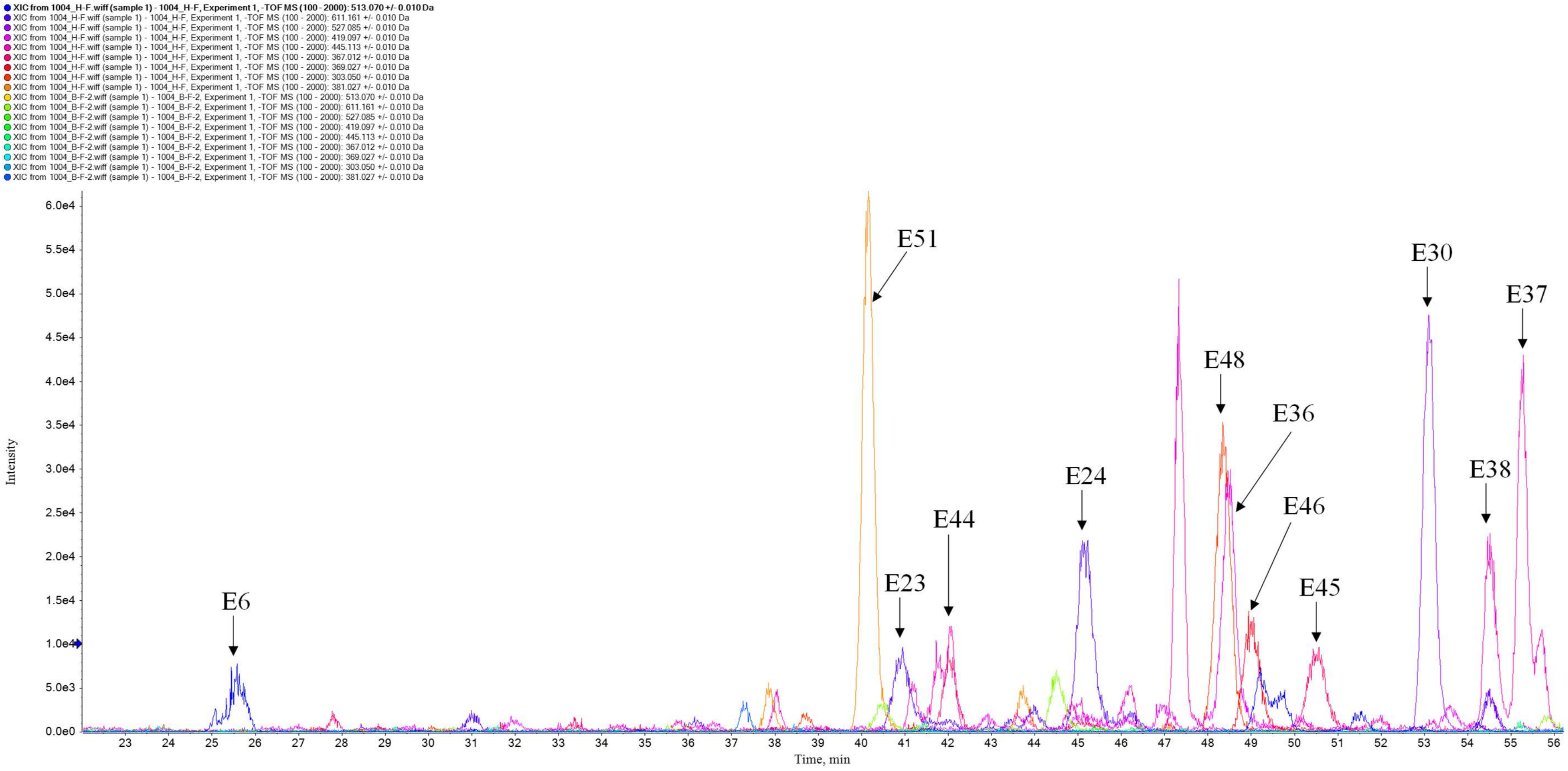

Figure S8. The EICs of compounds (E6, E23, E24, E30, E36–E38, E44–E46, E48, E51) in mice feces after administration of engeletin

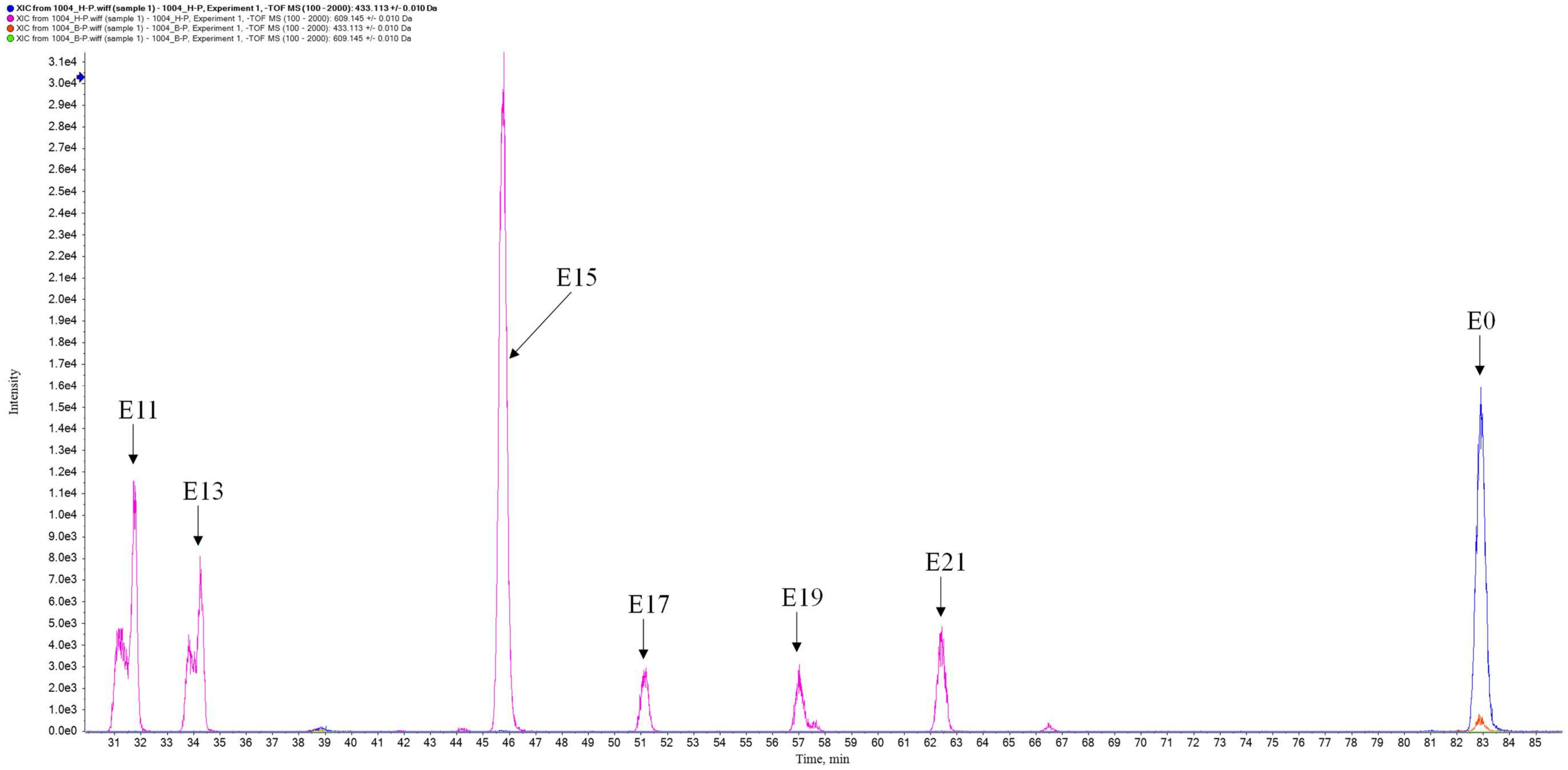

Figure S9. The EICs of compounds (E0, E11, E13, E15, E17, E19, E21) in mice serum after administration of engeletin

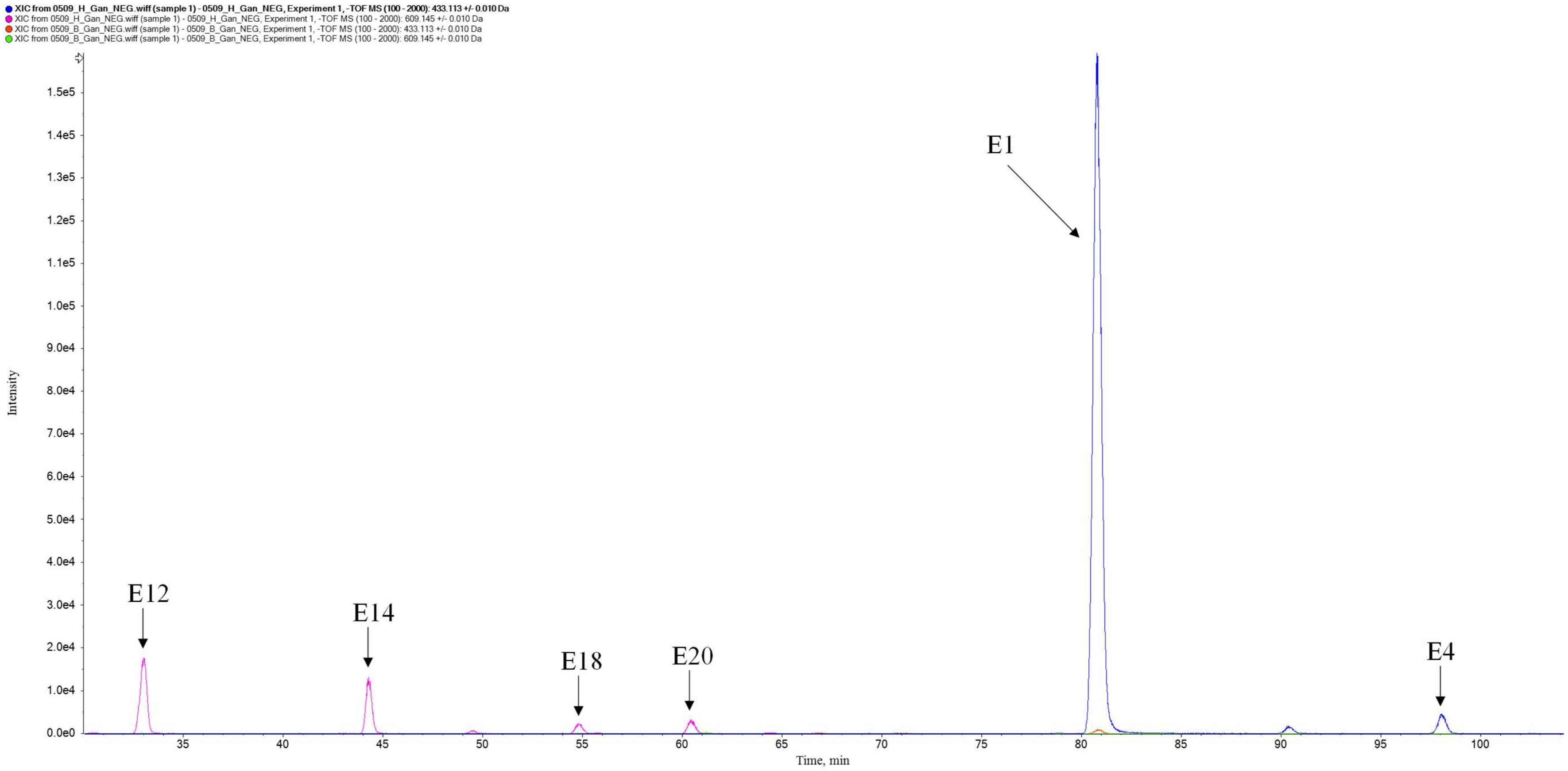

Figure S10. The EICs of compounds (E1, E4, E12, E14, E18, E20) in mice liver after administration of engeletin

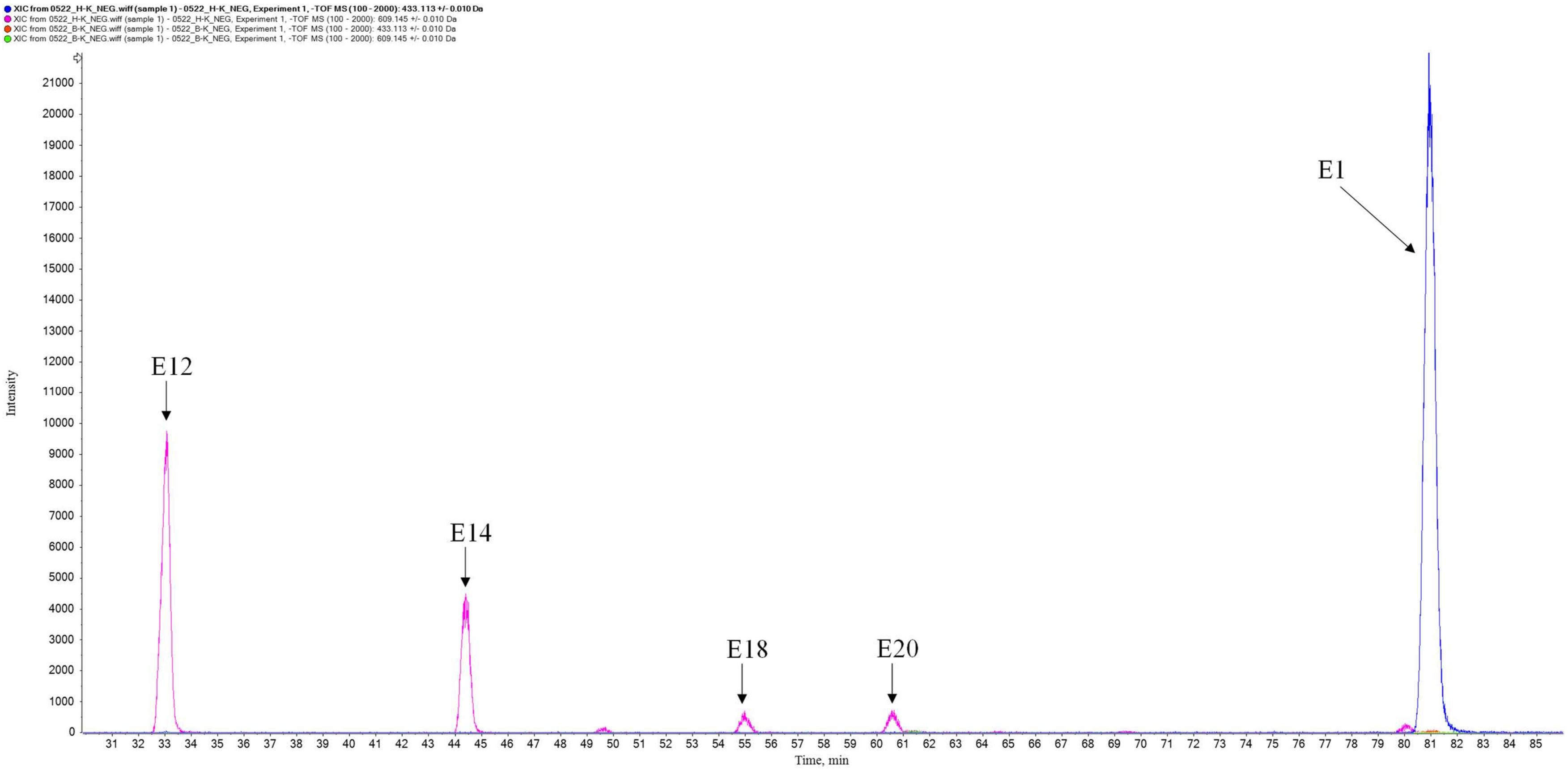

Figure S11. The EICs of compounds (E1, E12, E14, E18, E20) in mice kidney after administration of engeletin

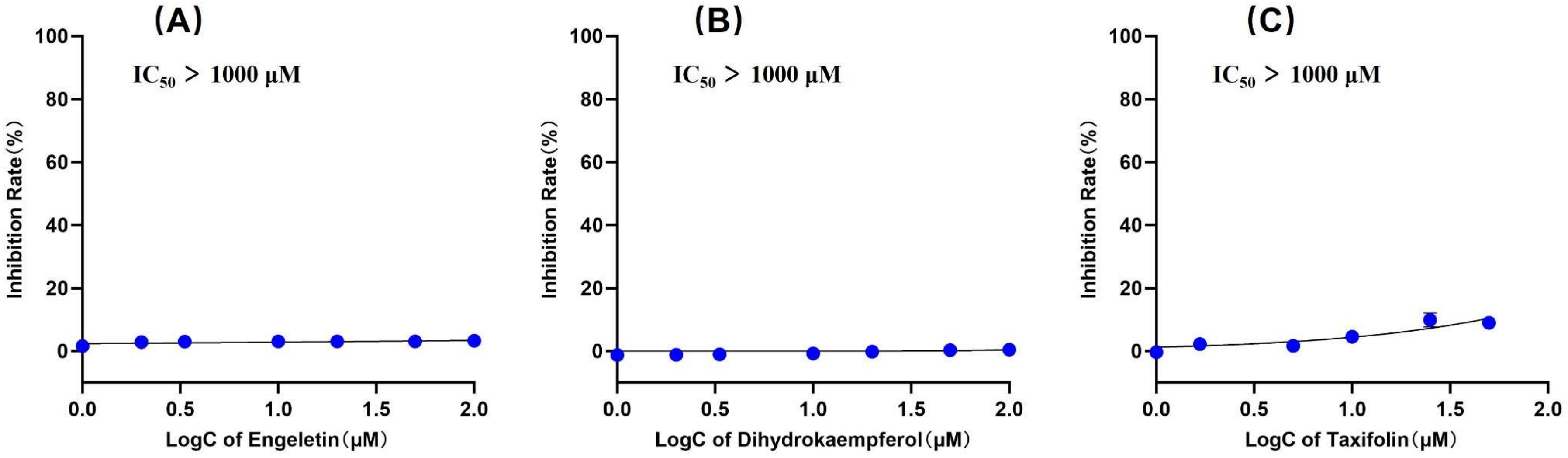

Figure S12. Inhibition rate of engeletin, dihydrokaempferol and taxifolin on xanthine oxidase. (A) engeletin; (B) dihydrokaempferol; (C) taxifolin. Data are represented as mean ± SD (n = 3).
